# Age-dependent cytokine surge in blood precedes cancer diagnosis

**DOI:** 10.1073/pnas.2420502122

**Published:** 2025-03-21

**Authors:** Guangbo Chen, Azam Mohsin, Hong Zheng, Yael Rosenberg-Hasson, Cindy Padilla, Kavita Y. Sarin, Cornelia L. Dekker, Philip Grant, Holden T. Maecker, Ying Lu, David Furman, Shai Shen-Orr, Purvesh Khatri, Mark M. Davis

**Affiliations:** ^a^Department of Microbiology and Immunology, Stanford University School of Medicine, Stanford, CA 94305; ^b^Department of Medicine, Stanford Center for Biomedical Informatics Research, Stanford University School of Medicine, Stanford, CA 94304; ^c^Division of Infectious Diseases, Department of Medicine, Stanford University School of Medicine, Stanford, CA 94304; ^d^Department of Dermatology, School of Medicine, Stanford University, Palo Alto, CA 94304; ^e^The Human Immune Monitoring Center, Stanford University, Palo Alto, CA 94304; ^f^Department of Biomedical Data Science, School of Medicine, Stanford University, Palo Alto, CA 94304; ^g^Buck Institute for Research on Aging, Novato, CA 94945; ^h^Stanford 1,000 Immunomes Project, Stanford School of Medicine, Stanford, CA 94305; ^i^Davis School of Gerontology, University of Southern California, Los Angeles, CA 90007; ^j^Department of Immunology, Faculty of Medicine, Technion Israel Institute of Technology, Haifa 3525422, Israel

**Keywords:** aging, cancer, immunology, cytokine surge

## Abstract

Aging is associated with increased variability and dysregulation of the immune system. We performed a system-level analysis of serum cytokines in a longitudinal cohort of 133 healthy individuals over 9 y. We found that cancer incidence is a major contributor to increased cytokine abundance variability. Circulating cytokines increase up to 4 y before a cancer diagnosis in subjects with age over 80 y. We also analyzed cytokine expression in 10 types of early-stage cancers from The Cancer Genome Atlas. We found that a similar set of cytokines is upregulated in tumor tissues, specifically after the age of 80 y. Similarly, cellular senescence activity and CDKN1A/p21 expression increase with age in cancer tissues. Finally, we demonstrated that the cytokine levels in serum can be used to predict cancers among subjects age at 80+ y. Our results suggest that latent senescent cancers contribute to age-related chronic inflammation.

## Significance

Many studies have demonstrated extensive and heterogeneous immune remodeling after cancer diagnosis in humans. However, we know little about how the human immune system interacts with transformed cells or tumors before a clinical diagnosis. In the Stanford-Ellison Cohort, we found that circulating cytokines increase years before a cancer diagnosis selectively in subjects age at 80+ y. We analyzed the cancer tissue transcriptome data from The Cancer Genome Atlas and found that the expression of the same set of cytokines also elevates in subjects age at 80+ y. These data suggest that senescent prediagnostic cancer tissues may contribute to systemic inflammation in an aged population.

Aging disrupts the homeostasis of immune system and increases its variability (1, 2). In a previous study on monozygous twins, we found that the variability of the immune system is mostly determined by nongenetic factors, with the variability increasing in an age-dependent manner (1). For example, the serum abundance of many cytokines (CXCL10, IL12p40, and others) is highly correlated between monozygous twins when they are young, but the correlation disappeared in the older group (60+ y) (1). These findings suggest that an age-associated process breaks the genetic and developmental constraint of immune phenotype. One of the well-known aging-associated processes is chronic inflammation (inflammaging) (3, 4). It is primarily attributable to cardiovascular risk factors, particularly obesity and inflammatory adipose tissues (5, 6). Beyond cardiovascular diseases, the incidence of a broad spectrum of other diseases also increases dramatically with age (7). For example, cancer incidence in people over 80 y old is 50 times higher than in people under 30 y old (National Cancer Registration and Analysis Service, England). Some of these age-associated diseases may perturb the immune system and increase its viability at the time of disease diagnosis or even during the latent phase.

We know very little about how the human immune system interacts with transformed cells or tumors before a clinical diagnosis. Many studies have demonstrated extensive and heterogeneous immune remodeling postcancer diagnosis in humans. Recent studies have also studied dynamic immune remodeling in detected and surgically resected early-stage lesions (8–12). However, it is unknown whether these local changes can be detected in the peripheral blood in a noninvasive manner. Years prior to a diagnosis, cancer is likely to be many orders of magnitude smaller than the one detectable by contemporary technology (~ 10^9 cells) (13). Whether the prediagnostic cancer tissue can trigger a measurable systemic immune response is an important question unanswered in the field.

In mice, studies have described some possible early systemic events in induced cancer models (14, 15). In young mice (typically 8 to 10 wk) with induced breast cancers, systemic immunity remodeling happens early (7 d after induction). Cytokines were found to orchestrate the remodeling of systemic immunity (14). However, the mouse model may not faithfully capture the biology of a spontaneous human cancer, which takes years or decades to form in people (16, 17). Meanwhile, the current mouse models do not fully represent the heterogeneity of age, cancer type, and genetic backgrounds intrinsic to a human population (2, 18, 19).

**Fig. 1.**
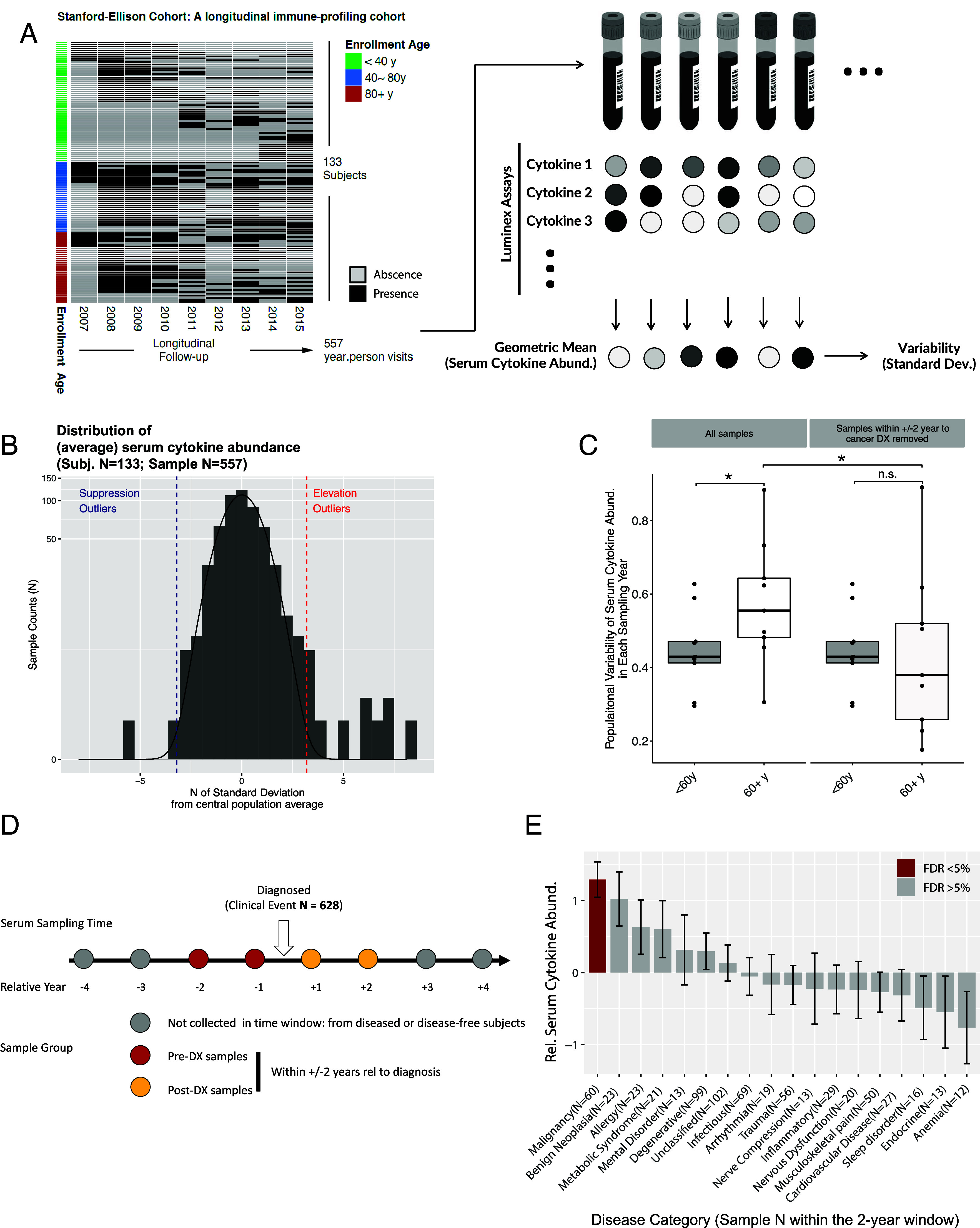
Elevated systemic cytokine levels correlate with cancer incidence. The data come from Stanford-Ellison Cohort. (*A*) The sampling and analytical scheme for a 9-y longitudinal cohort. (*B*) The distribution of geometric average cytokine abundance across 557 serum samples (referred to as serum cytokine abundance hereafter) collected in a 9-y longitudinal cohort is shown. Outliers are defined as samples with a significant deviation from the central populational mean (False Discovery Rate, FDR <0.05, *SI Appendix*). (*C*) The variability of serum cytokine abundances in different age groups is shown. Each point represents the SD among samples collected in a given sampling year for a given age group. Paired Wilcoxon rank tests were performed. n.s., *P* > 0.05, *, *P* < 0.05. (*D*) The scheme to examine the association between serum cytokine abundance and clinical events. The cytokine abundance was quantified within the defined time window (± 2 y relative to diagnosis). It was compared with samples collected outside the diagnosis time window. (*E*) The relative serum cytokine abundance for different disease categories. Label N provides the number of samples within the time window for a given category of diagnosis.

Many challenges, like sample availability, hinder the study of prediagnostic cancers in humans. Longitudinal studies in human cohorts, especially those focused on immunological biomarkers, can be invaluable. For this reason, we have used the Stanford-Ellison longitudinal cohort with its extensive immune profiling each year (20, 21). The age of participants ranges from 18 to over 90 y old. From 2007 to 2015, 557 annual visit serum samples were drawn from 133 participants (Fig. 1*A*, Demographics in *SI Appendix*, Table S1). At enrollment, the subjects were deemed “general good health and ambulatory” per inclusion criteria (*SI Appendix*). We assayed 32 cytokines in samples collected across years (**SI Appendix**). The clinical study team also collected in-depth medical histories from the participants.

We found that long-term serum cytokine levels rose up to 4 y before a cancer diagnosis (including nonmelanoma skin cancers/NMSC and other invasive cancers) and only in subjects 80 y and older. Similarly, in early-stage cancer tissues, we identified an inflammation phenotype that presents with an age threshold of 80 y. Finally, we demonstrated that the serum cytokine levels can be used to predict cancers among the subjects age at 80+ y.

## Results

### Cytokine Elevation Precedes a Cancer Diagnosis.

Cytokines in the blood work as messengers to coordinate downstream effectors, whose levels are sensitive to different perturbations. To characterize the systemic inflammation variation, we calculated the standardized geometric average abundance of 32 assayed cytokines (see *SI Appendix*, referred to as serum cytokine abundance below). There is considerable heterogeneity across samples (Fig. 1*B*). We found that serum cytokine abundance variability (population SD) increases with age (Fig. 1*C*). Seven cytokines (IP10/CXCL10, PDGFBB, IL-13, MCP3, IL-7, TNF-α, IL12p40) had significant increases of variability with age (FDR <0.05, *SI Appendix*, Fig. S1*A*). IP10/CXCL10 and IL12p40 were the top two cytokines whose serum concentration diverged during aging among a monozygous twin cohort we previously reported (1). Moreover, IP10/CXCL10 was reported to be the cytokine most significantly elevated by age, capable of predicting frailty (22). This increase in variability is not dependent on an overall increase of cytokine abundance in the subjects age at 80+ y, as the coefficient of variation (variability normalized by mean values) increases similarly as the unadjusted variability (*SI Appendix*, Fig. S1 *B* and *C*).

Aging is associated with a significant elevation of disease incidence, which may perturb the immune system (7). Of the 648 clinical events recorded during the 9-y follow-up, 628 (97%) occurred in people over 60. We hypothesized that the disease occurred around the sampling time might explain the variations of the systemic inflammation state. We asked whether the serum cytokine abundance altered within 2 y before or after a diagnosis (the diagnosis time frame) of a disease (Fig. 1*D*). We categorized 628 recorded clinical events into different categories (the category information for cancer, inflammatory conditions, and cardiovascular diseases in *SI Appendix*, Tables S2–S4). Among them, malignancies/cancer is the only one associated with a significantly changed (higher) level of serum cytokine abundances (Fig. 1*E*) (*SI Appendix*). Moreover, removing the samples collected within ± 2 y relative to a cancer diagnosis substantially lowers the variability of serum cytokine abundance among the old people (60+ y, Fig. 1*C*). Among the 7 cytokines whose variability significantly increased by age, cancer incidence is a major contributor (accounting for more than 50% of the increase) in 6 of them (*SI Appendix*, Fig. S1*A*). The coefficient of variation (variability normalized by mean values) also showed that cancer is a major contributor to the age-dependent increase of variance (*SI Appendix*, Fig. S1 *B* and *C*).

During the follow-up period, 46 cancer incidences were recorded (*SI Appendix*, Table S2). Similar to the cancer incidence rate in the US population [nonmelanoma skin cancers/NMSC5.4 million (23) vs. other cancers 1.6 million cases/year (24), the majority of the cancers were nonmelanoma skin cancers (33/46, *SI Appendix*, Table S2). Nonmelanoma skin cancers include basal cell carcinomas and cutaneous squamous cell carcinomas (cSCC), which are usually diagnosed at an early stage as a local disease. Nonmelanoma skin cancers share many cellular and genetic alterations with other cancers (25–28).

### Systemic Inflammation Occurs Before Cancer Diagnosis in an Age-Dependent Manner.

**Fig. 2.**
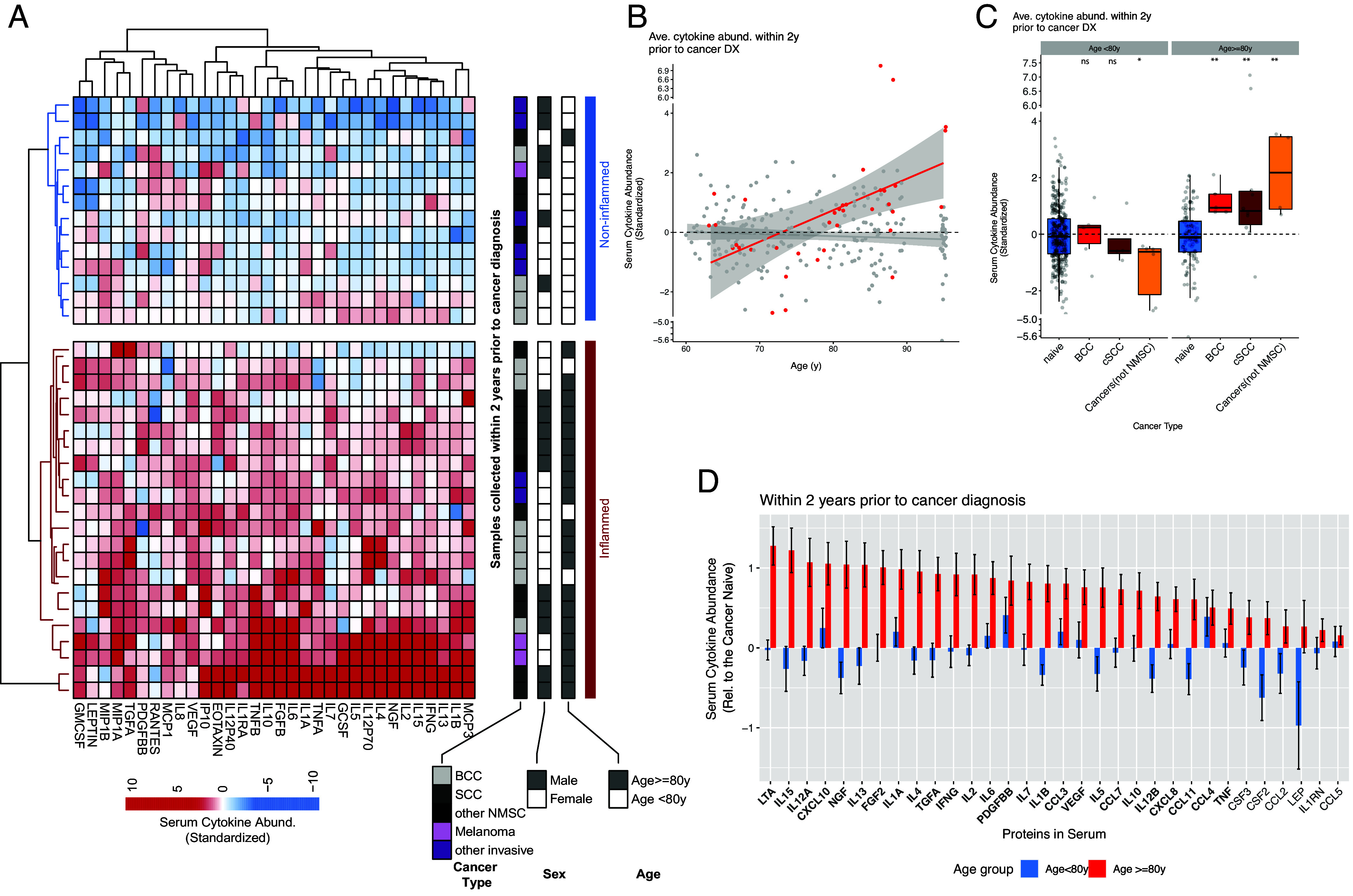
The level of a broad spectrum of cytokines rises in the blood samples collected before cancer diagnosis within the aged population (80+ y). The data come from Stanford-Ellison Cohort. (*A*) The heatmap of serum cytokine abundance in samples collected within 2 y before cancer diagnosis (prediagnostic serum samples). We used the Euclidean distance to cluster the samples and cytokines. The two major clusters of samples (inflamed vs. noninflamed state) are highlighted by colored sidebars (red vs. blue). In addition, three colored sidebars show the cancer types, sex, and sampling age. We examined the correlation between these clinical characteristics and the inflammation state. (*B*) The correlation between the serum cytokine abundance and age is shown. We examined the correlation in cancer naive subjects (without a cancer history, gray) or subjects going to have a cancer diagnosis within 2 y after the serum sampling (red). (*C*) The prediagnostic serum samples are grouped by cancer types. The serum cytokine abundance of samples is compared to the cancer naive subjects in each age group (<80 y or 80+ y). (*D*) The individual cytokine whose level rises in prediagnostic samples. Two age groups (<80 y and 80+ y) were compared to their matched (sex, age, and sample year, *SI Appendix*) cancer naive samples. The ones reached significance for the subjects age at 80+ y (FDR <0.05) are highlighted with a bold font. No cytokine reached significance in the younger group (all of their FDR >0.2).

We then examined whether the samples collected within 2 y before a cancer diagnosis show an increased level of cytokines (excluding postdiagnostic samples). Two distinctive cytokine response patterns emerged from the unsupervised clustering analysis: the inflamed vs. noninflamed (Fig. 2*A*, highlighted by red and blue sidebars). Individuals under the two patterns do not differ by sex or cancer type (Fig. 2*A*). However, the sampling age differs. Most noninflamed precancer diagnosis samples were from individuals less than 80 y old, while most of the samples showing a rise of cytokines were from those above 80 y old (1/10 vs. 11/14 subjects, odds ratio = 33) (Fig. 2*A*). The serum cytokine abundance strongly correlates with age only in samples collected within 2 y before a cancer diagnosis but not in the naïve group without a cancer diagnosis history (Fig. 2*B*). This correlation does not depend on the cytokine elevation outliers, suggesting that it is not solely driven by these individuals (*SI Appendix*, Fig. S2). This cytokine elevation depends on age regardless of cancer subtypes (Fig. 2*C*). In the following text, we call this phenomenon **pre-Cancer diagnosis Age-dependent Surge of cytokines (pre-CAS)** or just **cytokine surge.**

### A Broad Spectrum of Cytokines Elevates During the Surge.

A broad spectrum of cytokines elevates in precancer diagnosis samples from the subjects age at 80+ y (Fig. 2*D* red bars, cytokines with False Discovery Rate/FDR <5% highlighted by the bold font.), with all 32 assayed cytokines elevated and 26(81%) reaching FDR <5%(Including LTA, IL15, IL12A, CXCL10, NGF, IL13, FGF2, IL1A, IL4, TGFA, IFNG, IL2, IL5, PDGFBB, IL7, IL1B, CCL3, VEGF, IL5, CCL7, IL10, IL12B, CXCL8, CCL11, CCL4, TNF- α). In contrast, none are elevated (or changed) in subjects younger than 80 y (Fig. 2*D* blue bars, all of the cytokines with FDR > 20%). These pre-CAS cytokines represent a wide variety of functions. Some are proinflammatory (IL1, IL6, etc.), while others can be anti-inflammatory (IL-10). Some function primarily in innate immunity, while others participate in adaptive immune responses (IL2, interferon ɣ, etc.).

### Temporal Dynamics of the Cytokine Surge.

**Fig. 3.**
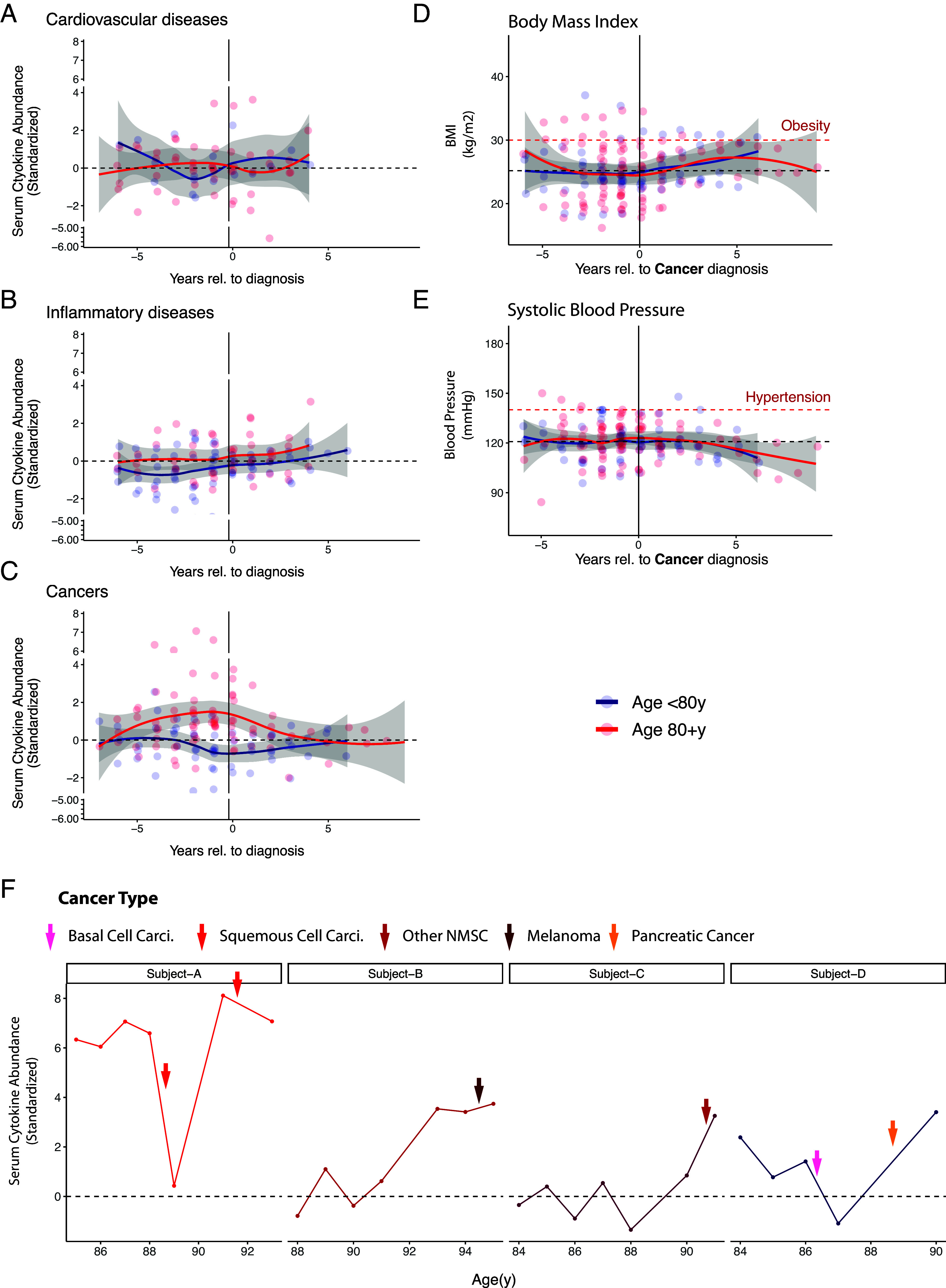
Long-term movement of serum cytokine baselines relative to disease diagnosis. The data come from Stanford-Ellison Cohort. (*A*–*C*) We plotted the serum cytokine abundance of samples collected around disease diagnoses (*A*, cardiovascular diseases; *B*, inflammatory conditions; *C*, cancers) on a time scale relative to the diagnosis. The samples are grouped by age (<80 y vs. 80+ y). The longitudinal profiles of subtypes of cancers or excluding the outliers are shown in *SI Appendix*, Fig. S3 *A–C*. The profiles for individual cytokine are shown in *SI Appendix*, Fig. S3*D*. (*D* and *E*) related to *C*, we plotted the two cardiovascular risk burdens (body mass index/BMI in *D*, and systolic blood pressure in *E*) against the sample collection times relative to cancer diagnosis. Upper limits of the normal values (BMI = 30, systolic blood pressure = 140 mmHg) are highlighted by the dashed red lines. The lines for smoothed average values and their 95-percentile CI in a-e were calculated by the loess function. (*F*) It shows the longitudinal profile of serum cytokine abundance of all subjects over 80 y with elevation outliers. All of them developed cancers, with diagnosis around the peak of cytokine abundance. Cancer type is indicated by color. The drop of cytokines at 89 y old for Subject A was verified by two independent samples in that year.

The multiyear longitudinal design of the Stanford-Ellison cohort allowed us to examine the long-term dynamics of the cytokine baseline relative to the timing of cancer diagnosis. This showed that the cytokine surge initiates up to 4 y before cancer diagnosis, but again, only in the subjects age at 80+ y (Fig. 3*C*, red line with the gray area of CI detached from the mean at −4 y), not those younger (<80 y, blue line). This is true for different cancer subtypes (*SI Appendix*, Fig. S3 *A* and *B*) and is not solely driven by the outliers (*SI Appendix*, Fig. S3*C*). Most individual cytokines behave similarly; the elevation starts years before cancer diagnosis (*SI Appendix*, Fig. S3*D*). Notably, the serum cytokines peak around the time of the clinical diagnosis and decrease thereafter, likely because of tumor resection. In mouse studies, it has been reported that tumor resection reversed changes in systemic immunity in the AT3 and 4T1 breast cancer and the MC38 colorectal cancer models (14). We also examined the longitudinal profiles of five individuals with cytokine elevation outliers (defined in Fig. 1*B*). Four (Subject A–D) of the five individuals are over 80 y old, all of whom have a cancer history. Cytokine surge concurred with cancer incidences (Fig. 3*F*).

In a cross-sectional cohort, Ferrucci et. al. measured 7 different cytokines and found that serum concentrations of 3 cytokines (IL6, IL1ra, IL18) increased linearly with age, with onset well below 80 y old. They also found that age-related chronic inflammation (“Inflamaging”) can largely be attributed to increased cardiovascular risk burdens (5). This type of age-associated increase of serum cytokines and its correlation with cardiovascular risk factors (including obesity and adipose tissues) was verified by many other studies (3–6). While the serum cytokine abundance rises prior to cancer diagnosis, the obesity (measured by body mass index) and systolic blood pressure do not (Fig. 3 *D* and *E*). In contrast to the temporal association with cancers, the serum cytokine abundance in this cohort does not change before or after the diagnosis of cardiovascular or inflammatory diseases (Fig. 3 *A* and *B*), potentially due to the limited flare duration of these diseases or a limitation of sample size of these events. The cancer-associated cytokine surge and the inflammation associated with cardiovascular risk burdens both correlate with age, and both are chronic inflammation. However, the two etiologies differ by the age onset and tissue origin.

### Augmented Inflammation in Early-Stage Cancer Tissues of the Subjects Age at 80+ y.

**Fig. 4.**
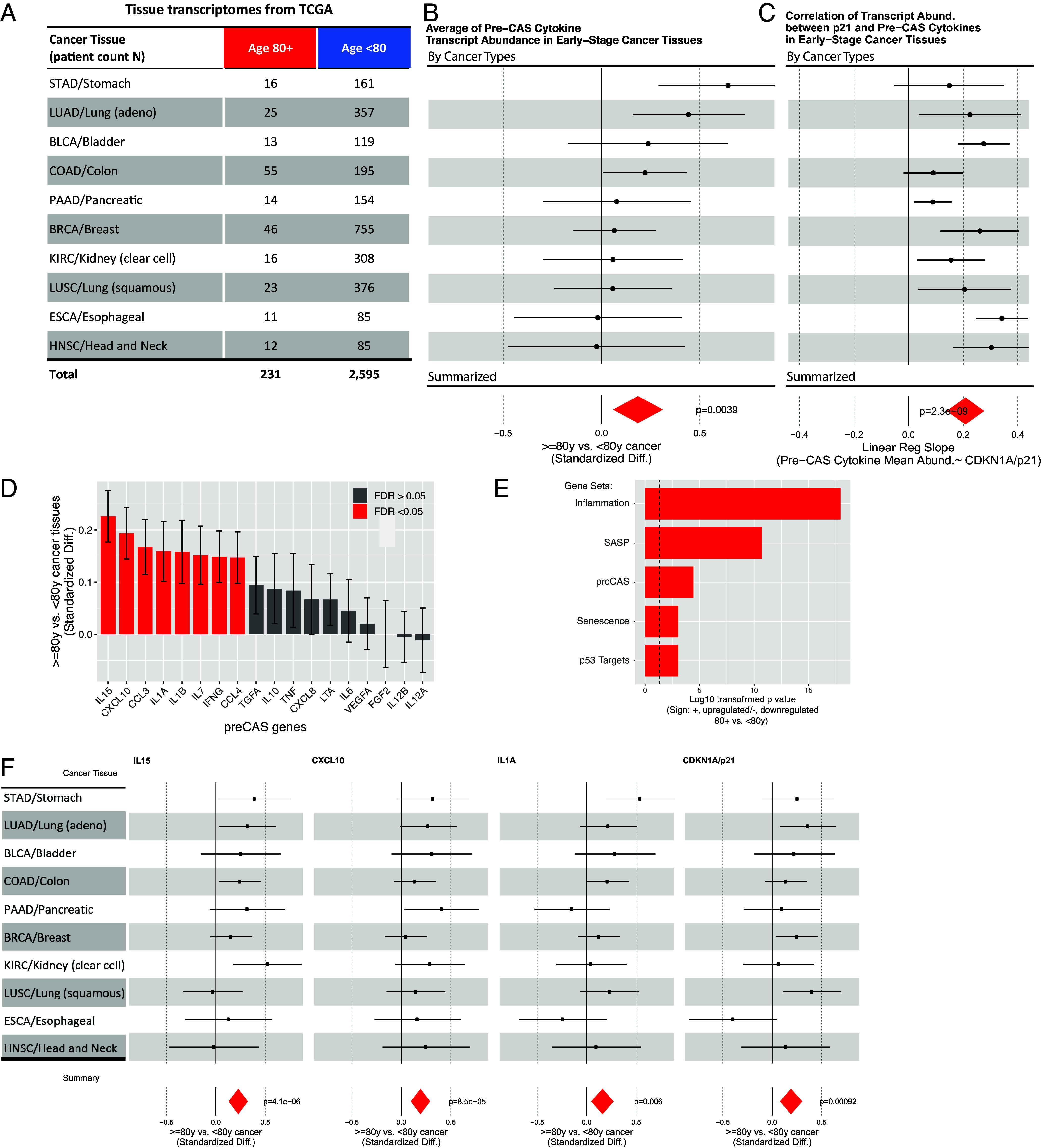
Advanced age elevates cytokine transcription in early-stage cancers along with senescence activity. The data come from TCGA. We examined the effect of advanced age (80+ y) on cytokine transcription (using the RNAseq dataset from TCGA) among early-stage cancer tissues across different cancer types (*SI Appendix*). (*A*) The sample counts for different age groups across cancer types are shown. (*B*) We calculated the geometric mean of transcript abundance of cytokine surge genes (Fig. 2*D*). This value is referred to as tissue cytokine abundance hereafter. The *Upper* panel reports the standardized difference/ effect sizes (defined in *SI Appendix*) for comparisons within individual cancer types. The length of the lines covers the 95 percentile CI, and the dot denotes the mean. The *Lower* panel reports the summarized standardized difference across cancer types with *P*-values. (*C*) similar to *B*, but we measured the correlation between the transcript abundance of CDKN1A/p21 and cytokine surge genes across early-stage cancers. (*D*) The standardized difference between <80 and 80+ y age groups are shown for individual cytokine surge genes. (*E*) We performed gene set enrichment analysis using the sets related to the cellular senescence process. *SI Appendix*, Table S7 provides the information for these gene sets. (*F*) Forest plots for cytokine genes and CDKN1A upregulated by age across cancer tissues. The presentation format is same to *B*.

These observations in peripheral blood suggest the inflammation activity in the cancerous tissues of the subjects age at 80+ y may be elevated. Therefore, we examined the transcriptional data for the early-stage (stage 1 to 2, local diseases) cancerous tissues in The Cancer Genome Atlas (TCGA), a large public RNAseq dataset (29). Ten cancer types, each with more than 10 samples from patients over 80 y old, were analyzed, covering 2,826 early-stage cancers (Fig. 4*A*, including 231 cases from 80+ y, see *SI Appendix*). In each sample, we focused on the cytokine genes relevant to the peripheral cytokine surge (Fig. 2*D*) and summarized transcription activity by taking a geometric average. The pre-CAS cytokine transcripts abundance in tissue is referred to as tissue cytokine abundance hereafter. Across cancer types, the older age group (80+ y) produces higher tissue cytokine abundance (Fig. 4*B* lower panel, *P* = 0.0039, adjusted for confounders, see *SI Appendix*).

To broadly measure the impact of the advanced age (80+ y) on the transcriptome of early-stage cancerous tissues, we extended our analytical pipeline similar to Fig. 4 *A* and *B* and examined the age-associated activity change of 50 hallmark gene sets (30). These gene sets were categorized into 7 different groups [among them, the immune-related group contains 9 gene sets, *SI Appendix*, Table S5, modified from (30). Compared with all other categories, the immune-related gene sets are particularly strongly upregulated, with every set highly significantly upregulated by the advanced age (FDR <1e-06, *SI Appendix*, Fig. S4*A* and Table S6). These gene sets cover a wide variety of immune-related processes, including the interferon response (α and γ) response, allograft rejection, TNFα signaling, etc. We also examined the cancer type–specific effect associated with the advanced age. Several previous studies have examined age-associated transcriptome changes across cancers. None of the studies distinguish early-stage cancers from late-stage ones, with a heterogeneous effect on immune-related gene sets among cancer types reported. However, we found immune-related gene sets upregulated across different cancer types in early-stage tissues (FDR<0.05 for 10/10 types for allograft rejection, 9/10 for interferon α and γ responses, *SI Appendix*, Fig. S4*B*). These results align with our findings that an advanced age (80+ y) associates with an elevation of cytokine production in the early-stage cancerous tissue (Fig. 4 *A* and *B*). Moreover, it underscores that immunity is the predominant process impacted by an advanced age in the early-stage cancerous tissues (*SI Appendix*, Fig. S4*A* and Table S6). Among the proteins involved in the serum cytokine surge (Fig. 2*D*), 18 were mapped onto the RNAseq dataset (*SI Appendix*). Sixteen of them showed positive values of age-associated cytokine transcription with 8 being significant (Fig. 4*D*). Among them, Interleukin-15 (IL15) stood out as a powerful activator for cytotoxicity in both Natural Killer cells and CD8+ T cells. IFNG, encoding for interferon γ, and its downstream effector, CXCL10, are also among the cytokines upregulated. Advanced age also increases the transcription of IL1A/B, two cytokines essential for launching inflammatory response (Fig. 4*F*). The substantial overlap between serum and tissue cytokine profiles supports the possibility that cancer tissue is a source of systemic inflammation.

### Advanced Age Is Associated with Increased SASP Activity and CDKN1A/p21 Expression.

Senescent cells accumulate in tissues during aging and secrete inflammatory factors, a phenomenon known as the senescence-associated secretory phenotype (SASP). The SASP can have both beneficial and detrimental effects, depending on the context (31–34). We analyzed the transcriptional activity of SASP genes across human cancers and found an age-dependent upregulation (35), which correlated with the age-dependent inflammation (Fig. 4*E* and *SI Appendix*, Table S7 for the gene lists of SASP and other senescence-related gene sets). Among the SASP factors that were upregulated by advanced age (80+ y), four (CXCL10/IP10, IL1A, IL1B, IL7) were also elevated in preCAS proteins (*SI Appendix*, Fig. S5*B*). In addition, markers of senescence (36) and primary p53 transcriptional targets (37) were also upregulated by advanced age (Fig. 4 *D* and *E*). One of the key regulators and markers of senescence, CDKN1A/p21, was upregulated by advanced age across cancer types (Fig. 4*F*). CDKN1A is a p53 transcriptional target required for SASP in cell line experiments (35, 38). In individual cancer types, the inflammatory gene sets (inflammation and preCAS) and senescence-related gene sets (senescence, SASP, p53 Targets) showed a concordant upregulation by age (*SI Appendix*, Fig. S5*A*).

We further examined whether the correlation between the senescence feature and cancer inflammation is independent of patient age. We found that the upregulation of CDKN1A/p21 transcripts is associated with a significant increase in the production of cytokines, and the association is uniform across cancer types (Fig. 4*C*). Moreover, the association remains strong if we only include patients under 80 (*SI Appendix*, Fig. S6). This observation suggests that patients can develop senescent and inflammatory cancers regardless of age.

### Age Correlates with Tissue Cytokine Abundance in a Nonlinear Manner.

**Fig. 5.**
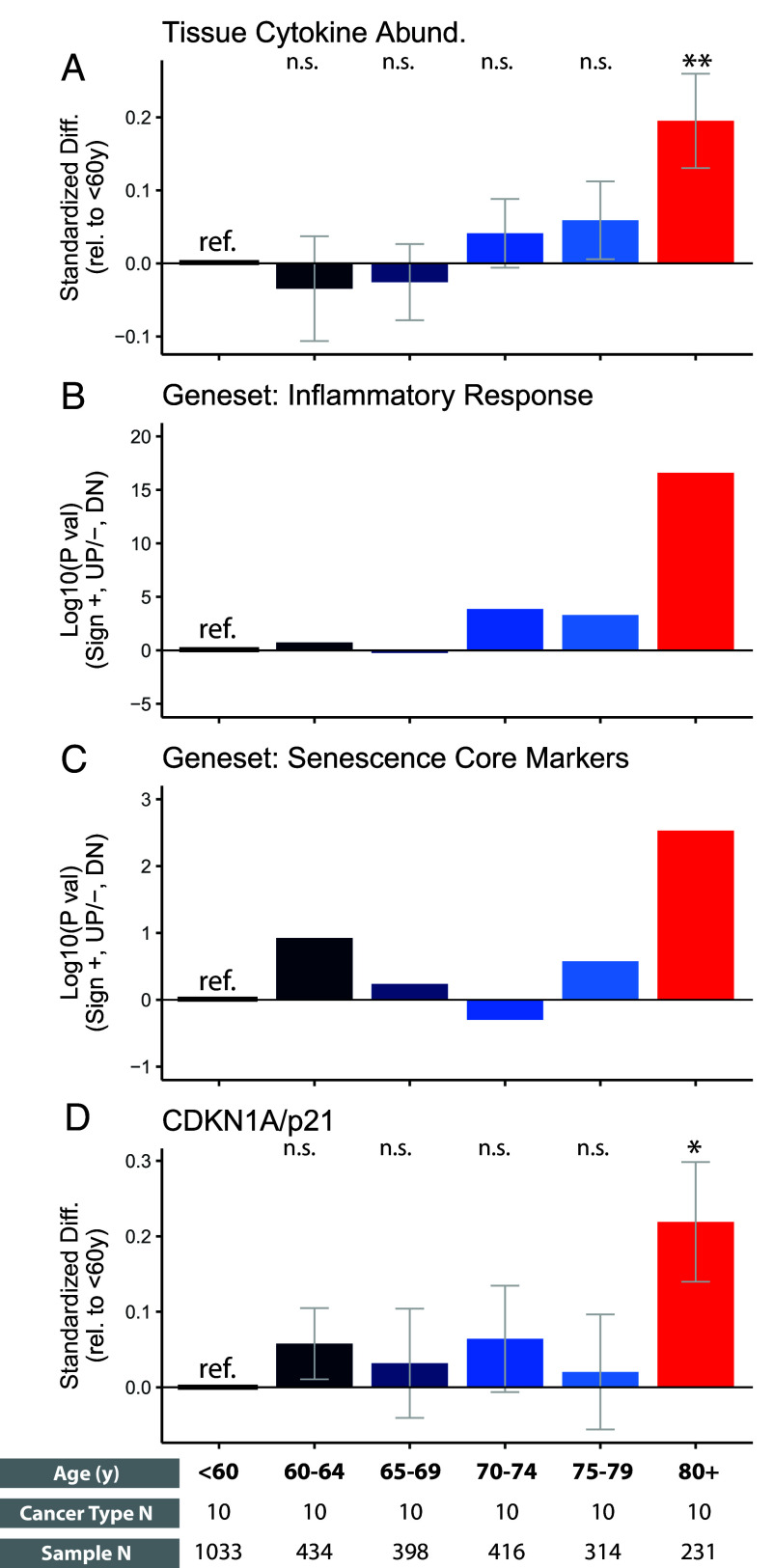
Tissue cytokine abundance correlates with age in a nonlinear manner. The data come from TCGA. We defined patients under 60 y old as the reference group. We compared all other 5-y age range subgroups with the reference group using a similar meta-analysis procedure, as shown in Fig. 4*B*. (*A*) Tissue cytokine abundance in different age groups is compared with the reference group. (*B* and *C*) We performed gene set enrichment analysis between different age groups and the reference group. The log10 *P* values are shown, with a sign indicating change directions (+, upregulation; −, downregulation). (*D*) The relative expression level of the CDKN1A gene in different age groups is shown. n.s., *P* > 0.05, *, *P* < 0.05; **, *P* < 0.01; ***, *P* < 0.001; ****, *P* < 0.0001.

Cytokine surge in the blood has an age threshold of 80 y. We wonder whether the age-dependent inflammation in cancer tissues shares the age threshold. We divided the samples into 5-y age groups, each containing hundreds of samples. We found that age correlated with tissue cytokine levels in a nonlinear manner, with most of the increase occurring only after 80 y old (Fig. 5*A*). Meanwhile, the activity of general inflammatory response correlated with age in a similarly nonlinear manner (Fig. 5*B*). The 80-y threshold also applied to the increase of cellular senescence activity and CDKN1A/p21 expression (Fig. 5 *C* and *D*). These results in tissue are concordant with our findings in the serum cohort (Fig. 3).

### Serum Cytokine Abundance Predicts Cancer Incidence in the Subjects Age at 80+ y.

**Fig. 6.**
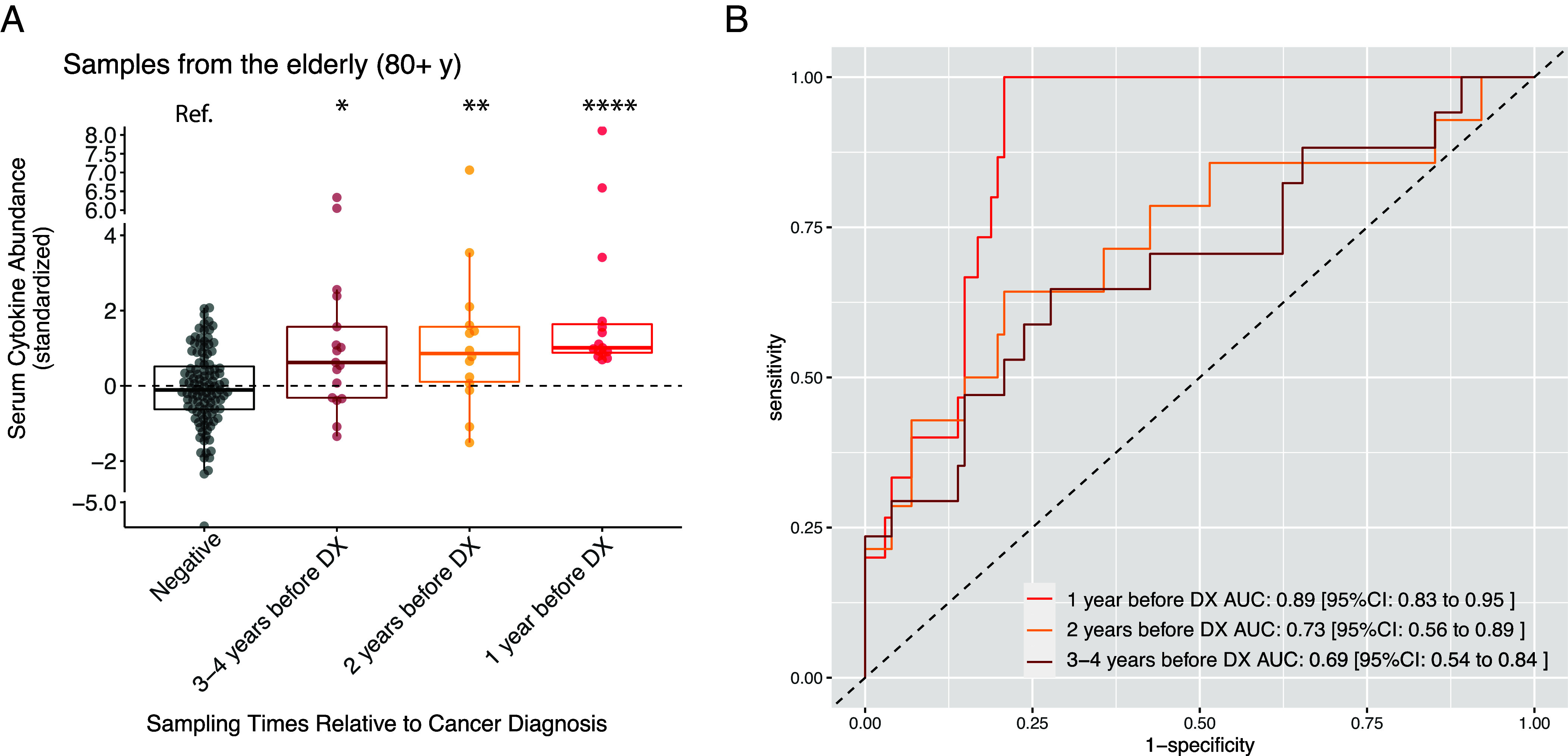
Serum cytokine abundance predicts future cancer incidence in the subjects age at 80+ y. The data come from Stanford-Ellison Cohort. The serum cytokine abundance is the geometric mean of 32 cytokines’ concentration in serum samples (same as Fig. 1*B*). (*A*) Samples from the aged group(80+ y) were grouped by the sampling time relative to the cancer diagnosis. All samples collected after the cancer diagnosis were removed. The negative samples include samples from cancer naive subjects as well as samples collected from cancer patients 5 or more years prior to the diagnosis. We performed Wilcoxon rank-sum test between the negative and other sample groups. n.s., *P* > 0.05, *, *P* < 0.05; **, *P* < 0.01; ***, *P* < 0.001; ****, *P* < 0.0001. (*B*) The ROC curve describes the accuracy of cancer prediction based on serum cytokine abundance. The comparisons were between the prediagnostic samples and the negative samples defined in *A*.

The aged population (80+ y) has a limited choice of therapeutics for cancer due to a heavy burden of comorbidities (39–41). The early diagnosis of cancers (potentially at a local disease stage) could allow less invasive therapeutic options. We tested whether the average serum cytokine abundance can be used for cancer prediction in the aged population. We found that the accuracy of prediction increases when the sampling time approaches the cancer diagnosis (Fig. 6*A*). The samples collected within 1 y prior to diagnosis (N = 15) can be distinguished from the noncancer samples (N = 111) with high accuracy [Area Under Receiver Operating Characteristic /AUROC = 0.89 (95 percentile CI: 0.83 to 0.95), Fig. 6*B*].

## Discussion

In search for the cause of why immune system becomes more variable by age, we found a remarkable age-dependent surge of cytokines up to 4 y before a cancer diagnosis in peripheral blood. Since cancers are generally much more treatable when caught early, a long-sought goal has been to develop detection methods that could warn of neoplasia much earlier. In recent years, significant advances have been made in early cancer diagnosis, based on technology detecting DNA or RNA molecules in the serum released from the cancer tissues (13). For example, the methylation signature for cell-free DNA can detect cancers with very high specificity, while the sensitivity is moderate [in the prespecified cancer types, sensitivity was 39% (CI: 27 to 52%) in stage I, 69% (CI: 56 to 80%) in stage II] (13, 42). Meanwhile, panels of protein markers, mostly derived from epithelial tissues (CA-125, CEACAM5, etc.), have also been shown to be valuable for early cancer detection (43, 44). Complement to these biomarkers, the immune system may function as an intrinsic signal amplifier to increase cancer detection sensitivity. It is critical to determine whether prediagnostic cancer tissues can trigger a systemic immune response detectable through noninvasive sampling and, if so, in whom, when, and how. Fortunately, we were able to draw on the long-running Stanford-Ellison longitudinal cohort (begun in 2007, following 133 participants for 9 y), which focuses on systems immunology profiling (20, 21). In our study, we found cytokine abundance can predict cancer incidence in the subjects age at 80+ y, while tumors from younger individuals appear to be more indolent in systemic cytokine activities prior to clinical diagnosis. In the future, the findings should be tested in a population with more heterogeneous population characteristics (such as ethnicity and health states) to examine the robustness. Compared with chronological age, biological age measured by various biomarkers can measure the aging state with a higher precision (45). It is interesting to test in a larger population whether some of the inflamed cancer patients with young chronological age demonstrate an old biological age. Meanwhile, our result does not rule out the possibility that immune monitoring [based on cytokines or other immune signaling factors, such as T cell repertoire (46, 47) may detect certain cancers prior to diagnosis in the younger population. In a mouse model, Wellenstein et. al. reported that the loss of p53 in breast cancers triggers systemic inflammation (48). Immune monitoring may complement other modes of early cancer detection technology and enhance accuracy.

We observed a gradual increase of cytokines in the years prior to cancer diagnosis in the subjects over 80 y old but a decrease after the diagnosis (and potentially resection) (Fig. 3*C*). The temporal dynamics suggest that the inflammation phenotype depends on the cancer development and is unlikely to come solely from noncancerous aging tissues. Our analysis of cancer tissue transcriptome further supports this model (Figs. 4 and 5). However, we also recognize that inflammation is well known to promote cancer development (49, 50). There is a possible vicious cycle between cancer development and inflammation in the years leading to the formation of cancer.

The striking age-dependence of cancer-associated cytokine surge in blood motivated us to inspect the cytokine production change among cancer tissues. We found that global cytokine gene transcription elevated in an age-dependent manner across cancerous tissues. Several previous studies have characterized immune-related transcriptome changes between samples collected from old and young cancer patients (51–53). However, there was considerable heterogeneity between different cancer types. Shah et al. reported an age-associated increase in immune response in lung squamous cell carcinoma but a decrease or no significant change in stomach, bladder, colon, and breast (52). Erbe et al. reported an age-associated increase in interferon gamma signaling among several cancer types (colon, lung, stomach, etc.) but decreased or no change among bladder and breast cancers (51). In our study, we identified the specific context and threshold for age to exert prominent effects on cytokine production. First, we found a consistent age-dependent elevation of cytokine production across different early-stage cancers (Fig. 4*B* and *SI Appendix*, Fig. S4*B*). In contrast, there is considerable heterogeneity among late-stage cancers (*SI Appendix*, Fig. S7), which may contribute to the heterogeneous findings among previous studies. Second, we found that the correlation between age and cytokine production is not linear, with the change concentrated in the aged (80+ y) group. Finally, previous age-association studies in cancer immunity only examined the local tissue. Our study found that systemic inflammation is age-dependent, with the same age cut-off of 80 y as in the cancer tissues (Fig. 5).

Finally, we found the age-dependent elevation of cellular senescence activity and CDKN1A/p21 expression in cancers. Using a transgenic mouse that permits tracking and eliminating senescent cells, Marco et al. showed that therapy-induced senescent cells in cancers could initiate systemic inflammation (54). It has been well documented that DNA damage repair efficiency declines with aging (55–57). For example, a mouse model with LacZ integrated into the genome showed a significant increase in mutant frequencies in the liver during aging (after 27 mo) (55). Similar findings have also been reported in human cells (58). The efficiency of DNA double-strand break repair by nonhomologous end joining and homologous recombination declines with aging (59, 60). The unrepaired DNA damage can trigger a sustained p53 activation, senescence (overexpression p21 and/or p16), and an inflammation phenotype (SASP) (61–63). For example, in mouse liver, p53-expressing senescent stellate cells release factors that skew macrophage polarization toward a tumor-inhibiting M1-state capable of attacking senescent cells in culture (62). Our results suggest that cellular senescence occurring naturally in the aging cancer tissue can cause local and systemic inflammation in humans.

Recent in vivo and ex vivo studies have demonstrated that senescent cells can activate innate and adaptive immunity, facilitating immunotherapy (64, 65). Meanwhile, clinical trials in human have demonstrated that immunotherapy works efficiently in the aged population despite a deteriorated immune system (66). In the future, it will be of interest to investigate whether cellular senescence is a therapeutic target for the tumors of the senior population.

## Materials and Methods

We conducted profiling of peripheral blood samples collected annually from young and older adults from 2007 to 2015 as part of the Stanford-Ellison longitudinal aging study. The cohort included 135 healthy individuals: 63 young adults (aged 20 to 31 at enrollment) and 72 older adults (aged 60 to 96 at enrollment). Written informed consent was obtained from all the study participants, and the study protocol was approved by the Stanford University Administrative Panels on Human Subjects in Medical Research (IRBs), confirming that the study complies with the relevant ethical regulations. Other details of the methods can be found in *SI Appendix*.

## Supplementary Material

Appendix 01 (PDF)

## Data Availability

Anonymized luminex data have been deposited in immport with accession numbers EXP34184 (67), EXP13427 (68), EXP13838 (69), EXP13809 (70), EXP11268 (71), EXP13837 (72), EXP13888 (73), EXP34260 (74), and EXP93748 (75).
