# Supplementary material for: Age-dependent cytokine surge in blood precedes cancer diagnosis
Source: Proc Natl Acad Sci U S A. 2025 Mar 21;122(12):e2420502122. doi: 10.1073/pnas.2420502122 (PMC11962427; doi:10.1073/pnas.2420502122)
Supplement: Supplementary file 1 — Appendix 01 (PDF) [file pnas.2420502122.sapp.pdf]

PNAS

Supporting Information for
Age-dependent Cytokine Surge in Blood Precedes Cancer Diagnosis

Guangbo Chen[1,2,3], Azam Mohsin[1], Hong Zheng[1,4], Yael Rosenberg-Hasson[1], Cindy Padilla[5], Kavita Y. Sarin[6], Cornelia L. Dekker[5], Philip Grant[5], Holden T. Maecker[1], Ying Lu[7], David Furman[8,9,10], Shai Shen-Orr[11], Purvesh Khatri[1,4], Mark M. Davis[1*]

[1]Institute for Immunology, Transplantation and Infection, Stanford University; Palo Alto, CA, 94304, United States.

[2]Current Address: Department of obstetrics and gynecology, Medical College of Wisconsin. Milwaukee, WI 53226, United States.

[3]Current Address: Center for Immunology, Medical College of Wisconsin. Milwaukee, WI 53226, United States.

[4]Center for Biomedical Informatics Research, Stanford University; Palo Alto, CA, 94304, United States.

[5]Stanford-LPCH Vaccine Program, Stanford University; Palo Alto, CA, 94304, United States.

[6]Department of Dermatology, School of Medicine, Stanford University; Palo Alto, CA, 94304, United States.

[7]Deartment of Biomedical Data Science, School of Medicine, Stanford University; Palo Alto, CA, 94304, United States.

[8]Buck Institute for Research on Aging; Novato, CA, 94945, United States.

[9]Stanford 1,000 Immunomes Project, Stanford School of Medicine, Stanford CA, 94305

[10]Austral Institute for Applied Artificial Intelligence, Institute for Research in Translational Medicine (IIMT), Universidad Austral, CONICET, Pilar, Buenos Aires, Argentina

[11]Department of Immunology, Faculty of Medicine, Technion Israel Institute of Technology; Haifa 3525422, Israel.

***Email:** mmdavis@stanford.edu

This PDF file includes:

Supporting text
Figures S1 to S7
Tables S1 to S5
SI References

Cohort Design

Peripheral blood samples were obtained at the Stanford Clinical and Translational Research Unit from a 9-year cohort; individuals were requested to return for annual visits after their year of enrollment, beginning in 2007(1).

All volunteers were assessed as generally healthy at the time of initial enrollment based on an evaluation of their medical history and assessment of their vital signs. Exclusion criteria at the time of enrollment included an active systemic or serious concurrent illness; a history of immunodeficiency; any known or suspected impairment of immunologic function; diabetes mellitus treated with insulin; moderate to severe renal disease; blood pressure greater than 150/95 at screening; chronic hepatitis B or C; recent or current use of immunosuppressive medication; malignancy other than squamous cell or basal cell skin cancer, including solid tumors such as breast cancer or prostate cancer with recurrence in the past year, and any hematologic cancer such as leukemia, which might jeopardize volunteer safety or compliance with the protocol; autoimmune disease; history of blood dyscrasias or hemoglobinopathies requiring regular medical follow up or hospitalization during the preceding year; use of any anticoagulation medication; a medical or psychiatric condition or occupational responsibilities that would preclude subject compliance with the protocol; history of Guillain-Barré syndrome; pregnancy; membership in the clinical study team; and any condition that might interfere with volunteer safety, study objectives or the ability of the participant to understand or comply with the study protocol. Development of these conditions at visits after enrollment did not exclude continuing participation in the study unless,

in the investigator's opinion, the condition interfered with the volunteer's safety, study objectives, or the ability of the participant to understand or comply with the study protocol. Whole blood was drawn and processed by standard procedures into peripheral blood mononuclear cells (PBMCs) and serum.

We conducted profiling of peripheral blood samples collected annually from young and older adults from 2007 to 2015 as part of the Stanford-Ellison longitudinal aging study. The cohort included 135 healthy individuals: 63 young adults (aged 20–31 at enrollment) and 72 older adults (aged 60–96 at enrollment)

Each year, the study physicians administered a standardized survey to update a participant's medical history and capture any new medical diagnoses. The disease incidences in recent years (not only the previous year) were recorded for the recruitment survey.

As the survey was performed annually, the temporal resolution of disease incidence is by year. For example, if we identified a subject diagnosed with cancer in the survey in the fall of 2009, the actual diagnosis was made between the 2008 and 2009 surveys. The blood samples collected in 2008 were labeled as (within) 1 year prior to cancer diagnosis.

Immune Profiling

The cytokines were measured using multiplexed liquid array bioassays (Luminex), using antibodies specific to each analyte. Due to the technology evolution (from 2007 to 2015), different variations of the Luminex platform were used for the measurement, with the number of measured cytokines varied. We

analyzed the 32 cytokines shared by all assays across the years. The detailed protocols are the following:

Luminex -EMD Millipore Human 80 Plex kits: This assay was performed by the Human Immune Monitoring Center at Stanford University- Immunoassay Team. Kits were purchased from EMD Millipore Corporation, Burlington, MA., and run according to the manufacturer's recommendations with modifications described as follows: H80 kits include 3 panels: Panel 1 is Milliplex HCYTA-60K-PX48. Panel 2 is Milliplex HCP2MAG-62K-PX23. Panel 3 includes the Milliplex HSP1MAG-63K-06 and HADCYMAG-61K-03 (Resistin, Leptin and HGF) to generate a 9 plex. The assay setup followed recommended protocol: Briefly: samples were diluted 3-fold (Panel 1&2) and 10-fold for Panel 3. 25ul of the diluted sample was mixed with antibody-linked magnetic beads in a 96-well plate and incubated overnight at 4°C with shaking. Cold and Room temperature incubation steps were performed on an orbital shaker at 500-600 rpm. Plates were washed twice with wash buffer in a BioTek ELx405 washer (BioTek Instruments, Winooski, VT). Following one-hour incubation at room temperature with biotinylated detection antibody, streptavidin-PE was added for 30 minutes with shaking. Plates were washed as described above and PBS was added to wells for reading in the Luminex FlexMap3D Instrument with a lower bound of 50 beads per sample per cytokine. Each sample was measured in duplicate. Custom Assay Chex control beads were purchased and added to all wells (Radix BioSolutions, Georgetown, Texas). Wells with a bead count <50 were flagged, and data with a bead count <20 were excluded.

Luminex -EMD Millipore Magnetic kits: This assay was performed by the Human Immune Monitoring Center at Stanford University. Custom 62- or 63-plex human cytokine kits were purchased from EMD Millipore Corporation, Burlington, MA., and used according to the manufacturer's recommendations with modifications described as follows. Briefly: samples were mixed with antibody-linked magnetic beads on a 96-well plate and incubated overnight at 4°C with shaking. Cold and Room temperature incubation steps were performed on an orbital shaker at 500-600 rpm. Plates were washed twice with wash buffer in a Biotek ELx405 washer. Following one hour incubation at room temperature with biotinylated detection antibody, streptavidin-PE was added for 30 minutes with shaking. Plates were washed as described and PBS was added to wells for reading in the Luminex FlexMap3D Instrument with a lower bound of 50 beads per sample per cytokine. Each sample was measured in duplicate. Custom Assay Chex control beads were purchased from Radix BioSolutions, Georgetown, Texas, and added to all wells.

Luminex -Polystyrene bead kits: This assay was performed in the Human Immune Monitoring Center at Stanford University. Human cytokine 37-, 50-, or 51-plex kits were purchased from Affymetrix and used according to the manufacturer's recommendations with modifications as described below. Briefly, samples were mixed with antibody-linked polystyrene beads on 96-well filter-bottom plates and incubated at room temperature for 2 h followed by overnight

incubation at 4°C. Room temperature incubation steps were performed on an orbital shaker at 500-600 rpm. Plates were vacuum filtered and washed twice with wash buffer, then incubated with biotinylated detection antibody for 2 h at room temperature. Samples were then filtered and washed twice as above and resuspended in streptavidin-PE. After incubation for 40 minutes at room temperature, two additional vacuum washes were performed, and the samples re-suspended in Reading Buffer. Each sample was measured in duplicate. Plates were read using a Luminex 200 instrument with a lower bound of 100 beads per sample per cytokine. Custom assay Control beads by Radix BioSolutions are added to all wells.

Data pre-processing

We used R running on Rstudio to perform all statistical analyses.

To eliminate the year-to-year batch effect, we use ComBat(2) method in the SVA package(3). The method is based on an empirical Bayes method. A previous study found that the participants under 40 years old demonstrated high overall longitudinal stability for immune phenotypes(1). Thus, we assumed the young people (under 40y) would have similar mean values and variations for the serum cytokine abundance across years and used the population as "anchor points" across years to remove the year-to-year batch effect.

Standardize the serum cytokine abundance

To summarize the serum abundance of 32 cytokines assayed across the years, we calculated the geometric mean. Compared with the arithmetic mean, the geometric mean is more robust and not prone to dominance by high-abundance cytokines.

As shown in Figure 1b, while the average cytokine abundance across samples largely followed a normal distribution, outliers existed. To reduce the impact of outliers in estimating the standard deviations (**SD**) of the population, we calculated the standard deviations using only the central population (quantile 25%- 75%)(1):

First, we use the central population to calculate a raw SD.

Secondly, we corrected the raw SD by multiplying a correction factor (2.65). The correction is necessary as the central population is truncated by 25-75 percentiles, so the raw SD represents an underestimation of the true SD. The correction factor is derived by comparing the SD between a simulated full normal distribution and a truncated 25-75 percentile population.

Analyze the variability of cytokine abundance in different age groups

For each sampling year, we calculated the standard deviation and coefficients of variation (standard deviation normalized by mean) of cytokine abundances among two age groups: 60+ vs. <60 years. Between-group analysis was performed using the Wilcoxon rank test, paired by year.

Correlate the serum cytokine abundance with disease incidences

As described in Figure 1d the serum cytokine abundance was quantified within the defined time window (+/- 2 years relative to diagnosis), compared with the one from all samples collected outside the time window. Because vast majority of the disease incidences happening in the seniors (> 60 years old), we only included that population.

We included the age and sex as covariates in the linear regression model to estimate the disease-specific effects (adjusted difference, Figure 1e).

Correlate the individual cytokine abundance with cancer diagnosis

All cancer incidences in the cohort happened in subjects over 60 years old. All following analysis includes only subjects over 60 years old. The pre-diagnostic samples are defined as the samples collected within 2 years prior to the 1st cancer diagnosis. Cancer naïve subjects are defined as subjects without a recorded cancer incidence during the follow-up period (the median follow-up time for cancer naïve subjects is 7 years, [quantile range 4.8- 8 years], which is similar to the subjects with a cancer history. See Extended Data Table 1). The unsupervised clustering analysis identified two inflammation states of pre-diagnostic samples, with the inflamed samples nearly exclusively from the elderly (80+ y, Figure 2a).

We separated the pre-diagnostic samples (within 2 years prior to cancer diagnosis) into 2 groups: <80 years and 80+ years (the elderly). To avoid the confounding by

sex and age differences, we matched each pre-diagnostic sample to two samples collected from cancer naïve subjects. The matching was performed using the propensity score(4), which accounts for the confounders in a probabilistic model. R package matchit was used for the matching(5).

A multivariate linear regression model (LIMMA) including sex, age, collection batch, and propensity scores was used to estimate the serum cytokine abundance difference associated with samples collected prior to cancer diagnosis(6). The combination of matching and multivariate linear regression aimed to reduce related confounding effects maximumly.

TCGA/cancer tissue transcriptome data pre-processing

Gene expression data of the TCGA cohort was obtained from Synapse (ID syn4976369, link: https://www.synapse.org/#!Synapse:syn4557014). It contains 20531 genes and 11069 samples processed with RSEM. Genes were adjusted for batch effect using a novel algorithm called EB++, a variant of the Empirical Bayes / ComBat algorithm with training and testing features added. Genes with NA values were removed.

Measure the mean difference of cytokine abundance associated with aging in cancer tissues

We hypothesized that the early-stage pre-diagnostic cancer tissues might be the source of the cytokine surge in the blood. We focused our analysis on the cancer

tissues from the local diseases (stages 1-2). Advanced-stage (stages 3-4) cancers were also analyzed separately for comparison (Figure S7).

Within the 33 types of cancers collected by The Cancer Genome Atlas (TCGA)(7), we chose the 10 types of cancers where there are more than 10 samples from the elderly (80+ y), including stomach, lung adenocarcinoma, bladder, colon, pancreatic, breast, kidney (clear cell), lung squamous, esophageal, head and neck cancers. In total, 2,926 early-stage cancer samples cancer samples were included.

In the blood, there are 26 cytokines whose abundance significantly elevates within 2 years prior to a cancer diagnosis in the elderly (Figure 2d, **pre-Cancer diagnosis Age-dependent Surge of cytokines (pre-CAS)**). To reflect the tissue cytokine transcription activity relevant to our findings in the blood, we calculated the geometric mean of pre-CAS cytokines' mRNA abundances in each individual sample (referred to as **Tissue Cytokine Abundance** hereafter).

For each cancer type, we measured the transcript abundance difference between the elderly and the younger population. We used a linear regression model to account for the confounding factors, including sex and stage. To make the difference comparable between different cancer types, we standardized the tissue cytokine abundance within each cancer type (Scaled so that the standard deviation equals to 1).

Across the cancer types, the summary effect size g_s is computed using a random effect model as:

$$g_s = \frac{\sum_i^n W_i g_i}{\sum_i^n W_i}$$

where Wi is a weight equal to $1/(Vi+T^2)$, where Vi is the variance of that gene within a given cancer type i, and T^2 is the inter-cancer type variation.

We used the R package rmeta and data.table to implement the meta-analysis procedure(8).

Pathway analysis for transcriptome changes associated with age and genotypes

The original Molecular Signature Database included over 10,000 gene sets. The 50 hallmark gene sets represent "refined" gene sets that summarize most of the relevant information of the original founder sets and, by reducing both variation and redundancy, provide more refined and concise inputs for gene set enrichment analysis(9). These gene sets cover a wide variety of biological processes, categorized into 7 different groups (Table S5, modified from (9)).

To examine the enrichment of age-associated gene expression alterations within each gene set, we use the Generally Applicable Gene-set Enrichment (GAGE), an R package for gene set analysis based on a parametric gene randomization procedure(10). In the analysis, we used the default settings of the program.

Figure S1: Age-associated variability change of individual serum cytokine protein
The data come from Stanford-Ellison Cohort. We measured the serum cytokine abundances for 133 participants over a 9-year follow-up period. **a)** In each year, we calculated the variability (standard deviation) for the two-age group (<60 and 60+ years). The difference is shown in the figure. The black bar represents the comparison using all samples, while the grey bars represent the comparison using only samples not within the +/-2 years relative to a cancer diagnosis. The error bar represents the standard error derived from the 9 year replicates. The top 2 cytokines whose concentration diverges in the old (60+ years) mono-zygous twin cohort previously reported are highlighted by red. The star represents False Discovery Rate (FDR) < 0.05. **b)** Similar to **Figure 1c**, but we calculated the coefficients of variations for each sample group. The coefficients of variation represent the standard deviation normalized by mean values. **c)** Similar to **a**, but we calculated the coefficients of variations for each sample group for each cytokine.

FigureS2: Average cytokine abundance correlates with age in samples collected prior to cancer diagnosis (cytokine elevation outliers excluded)
The data come from Stanford-Ellison Cohort. The figure is related to **Figure 2b**. The cytokine elevation outliers are defined in **Figure1b**. The upward trend remains after we removed all 4 cytokine elevation outliers (> 3 standard deviations from the central population mean) within 2 years prior to a cancer diagnosis.

Figure S3: Long-term movement of serum immune baseline relative to disease diagnosis This figure is related to **Figure 3c**.
The data come from Stanford-Ellison Cohort. The average cytokine abundance in serum samples collected around cancer diagnosis is displayed. Various subgroups were examined to test the robustness of the finding. **a-b)** Cancers were subdivided into non-melanoma skin cancers and other cancers. **c)** Cytokine elevation outliers were removed to examine the robustness of the cytokine surge around cancer diagnosis in the subjects age at 80+y. Samples are grouped by age (<80y vs. 80+ y, blue vs. red lines). **d)** Long-term movement of individual serum cytokine abundance relative to cancer diagnosis is shown. Samples are grouped by age (<80y vs. 80+ y). Arrowheads mark the earliest time point at which the moving average elevates above 1 standard deviation, and the time point is displayed as a number (years).

Figure S4: Gene set enrichment analysis shows the impact of the advanced age on the transcriptome of early-stage cancer tissues.
The data come from TCGA. The gene set analysis used 50 hallmark gene sets covering a wide variety of biological functions with a reduced level of overlapping between sets compared to the whole gene sets(30). **a)** Gene set enrichment analysis on the age-dependent transcriptome changes shared across 10 cancer types (as described in **Figure 4a**). The gene sets are grouped into 7 functional

categories (**Table S5**). Log10 transformed False Discovery Rates were reported. Each point represents the age-dependent change of a given gene set, with the top-up-regulated immune gene sets marked out by texts. **b)** Gene set enrichement analysis for expression change associated with advanced age in early-stage cancer is shown for each included cancer type. The heatmap is clustered by Euclidean distance on both rows (gene sets) and columns (cancer types). Star denotes the gene sets significantly changed in advanced age (80+y, FDR <0.05).

Figure S5: Senescence and SASP gene sets activities are upregulated by age across cancers.
The data come from TCGA. a) Gene set analysis in different cancer types demonstrates concordant upregulation of inflammatory gene sets (inflammation, preCAS) and senescence-related gene sets (senescence, SASP, p53 transcriptional targets). The log10 transformed p-value is reported with the sign indicating the age-dependent changes. b) The four SASP genes whose transcripts are upregulated by age in cancer tissues. Note these 4 proteins are also upregulated by cytokine surge in serum samples collected prior to cancer diagnosis (**Figure 2d**).

Figure S6: Correlation between the transcript abundance of CDKN1A/p21 and cytokines in subjects age under 80y.
The data come from TCGA. The figure is similar to **Figure 4c** but the correlation is performed only among subjects age under 80y.

Figure S7: The age-dependent inflammation is specific to early-stage cancers
The data come from TCGA. The figure is similar to **Figure 4b** but adds comparisons between late-stage cancers (stages 3-4). We calculated the geometric mean of transcripts abundance of cytokine surge genes (**Figure 2d**), defined as tissue cytokine abundance. We compared the tissue cytokine abundance between age groups (80+y vs. <80y) in each cancer type (by row) and each stage group (early vs. late). The standardized differences/ effect sizes (defined in supplementary methods) are reported in the upper panel. The length of the lines covers the 95-percentile confidence intervals, and the dot denotes the mean. The summarized standardized difference is reported in the lower panel with p-values (supplementary methods).

Figure S1

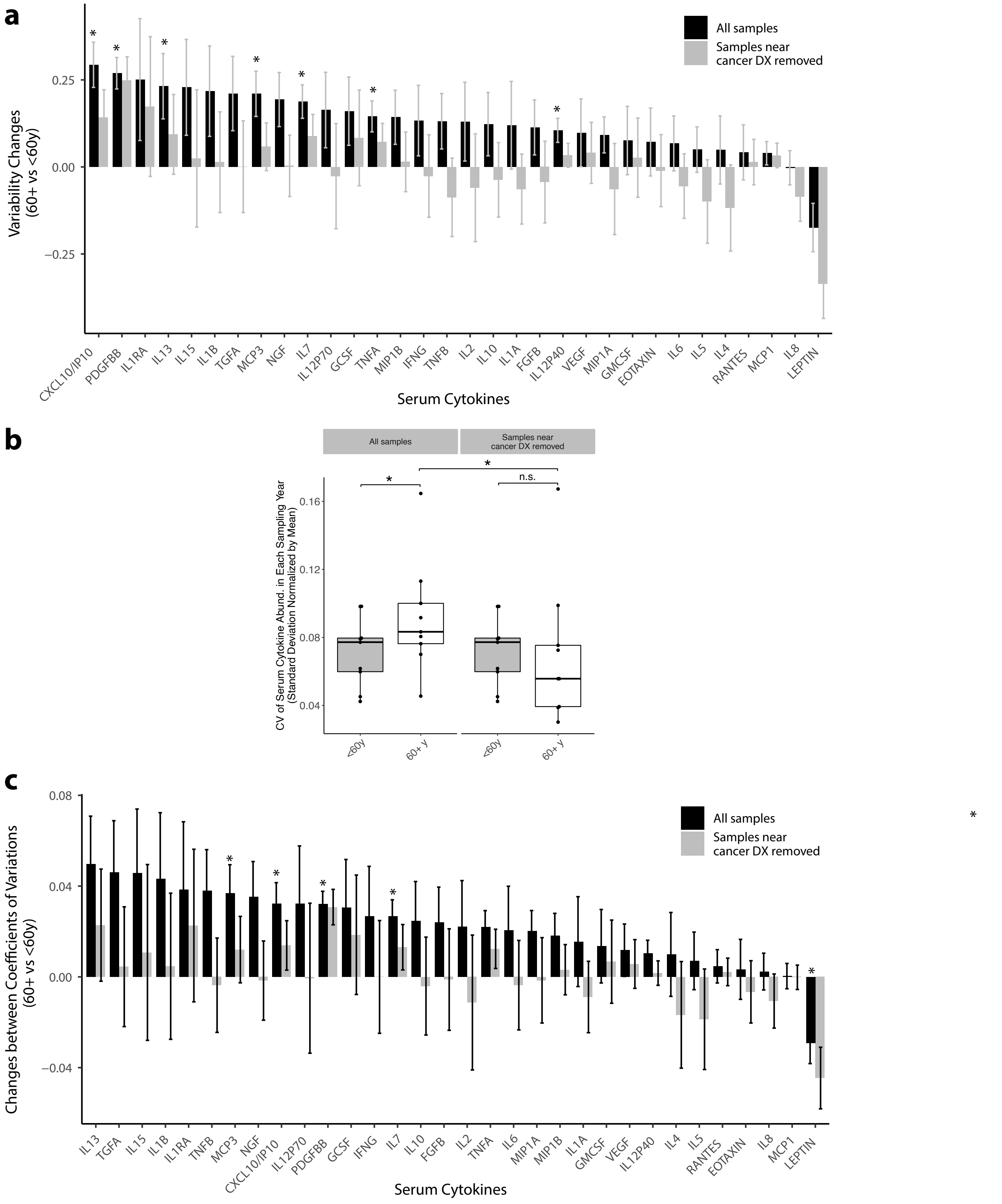
a
All samples
Samples near cancer DX removed
Variability Changes (60+ vs <60y)
0.25
0.00
−0.25
CXCL10/IP10 PDGFBB IL1RA IL13 IL15 IL1B TGFA MCP3 NGF IL7 IL12P70 GCSF TNFA MIP1B IFNG TNFB IL2 IL10 IL1A FGFB IL12P40 VEGF MIP1A GMCSF EOTAXIN IL6 IL5 IL4 RANTES MCP1 IL8 LEPTIN
Serum Cytokines
b
All samples
Samples near cancer DX removed
n.s.
CV of Serum Cytokine Abund. in Each Sampling Year (Standard Deviation Normalized by Mean)
0.16
0.12
0.08
0.04
<60y
60+ y
<60y
60+ y
c
All samples
Samples near cancer DX removed
Changes between Coefficients of Variations (60+ vs <60y)
0.08
0.04
0.00
−0.04
IL13 TGFA IL15 IL1B IL1RA TNFB MCP3 NGF CXCL10/IP10 IL12P70 PDGFBB GCSF IFNG IL7 IL10 FGFB IL2 TNFA IL6 MIP1A MIP1B IL1A GMCSF VEGF IL12P40 IL4 IL5 RANTES EOTAXIN IL8 MCP1 LEPTIN
Serum Cytokines

Figure S2

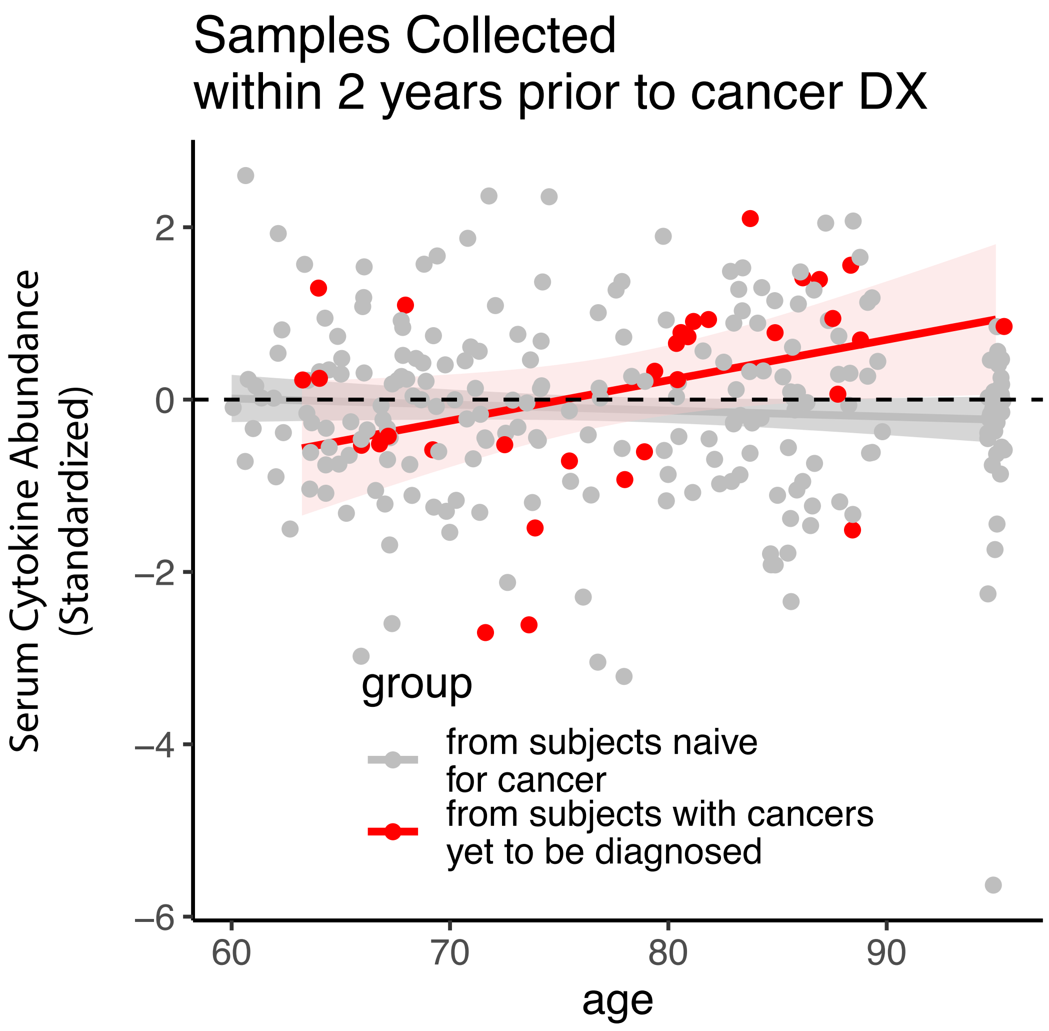
Samples Collected
within 2 years prior to cancer DX
Serum Cytokine Abundance
(Standardized)
2
0
−2
−4
−6
60
70
80
90
age
group
from subjects naive
for cancer
from subjects with cancers
yet to be diagnosed

Figure S3

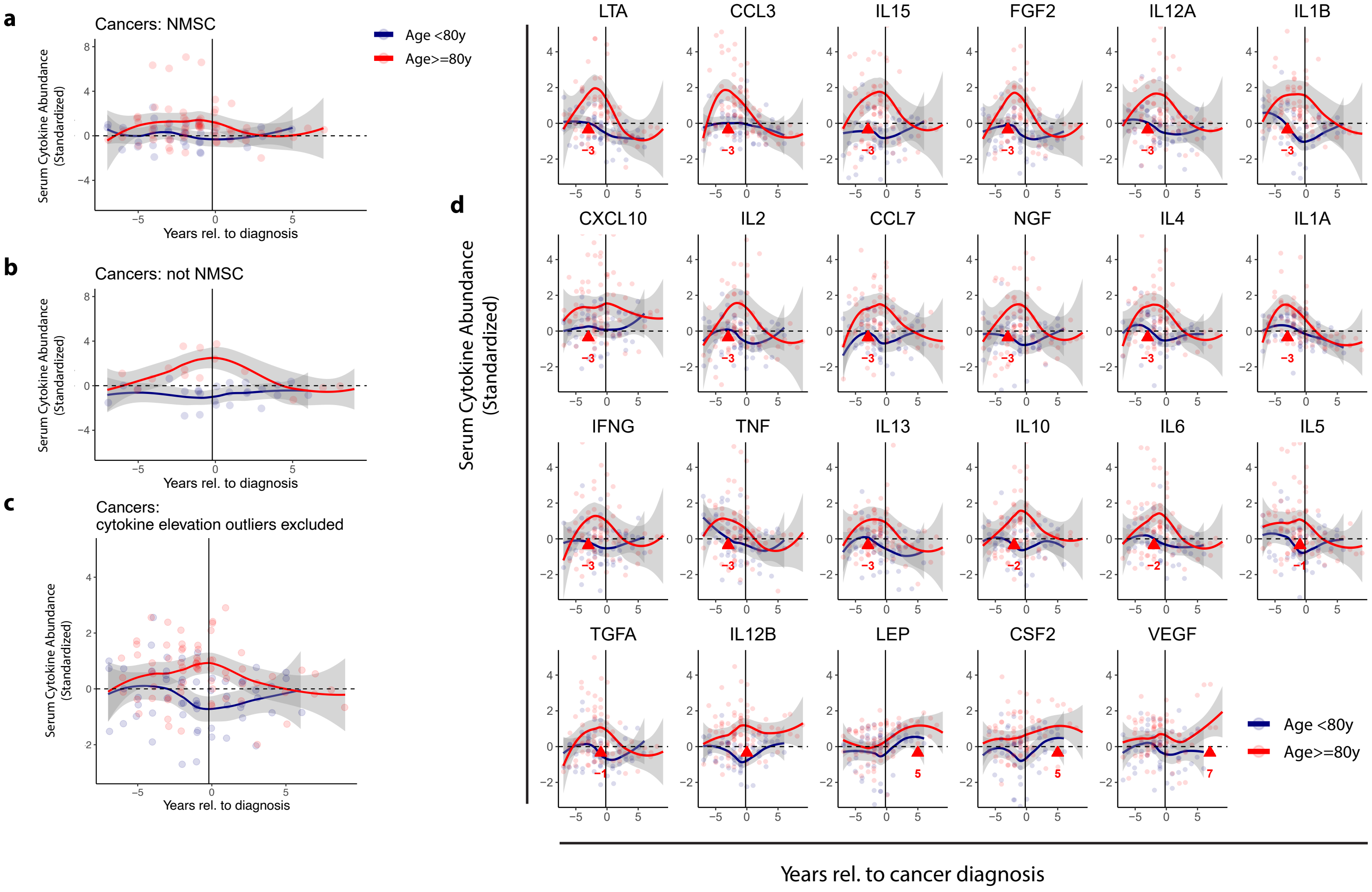

a
Cancers: NMSC
Serum Cytokine Abundance (Standardized)
Years rel. to diagnosis
Age <80y
Age>=80y
b
Cancers: not NMSC
Serum Cytokine Abundance (Standardized)
Years rel. to diagnosis
c
Cancers:
cytokine elevation outliers excluded
Serum Cytokine Abundance (Standardized)
Years rel. to diagnosis
d
Serum Cytokine Abundance (Standardized)
LTA
CCL3
IL15
FGF2
IL12A
IL1B
CXCL10
IL2
CCL7
NGF
IL4
IL1A
IFNG
TNF
IL13
IL10
IL6
IL5
TGFA
IL12B
LEP
CSF2
VEGF
Age <80y
Age>=80y
Years rel. to cancer diagnosis

Figure S4

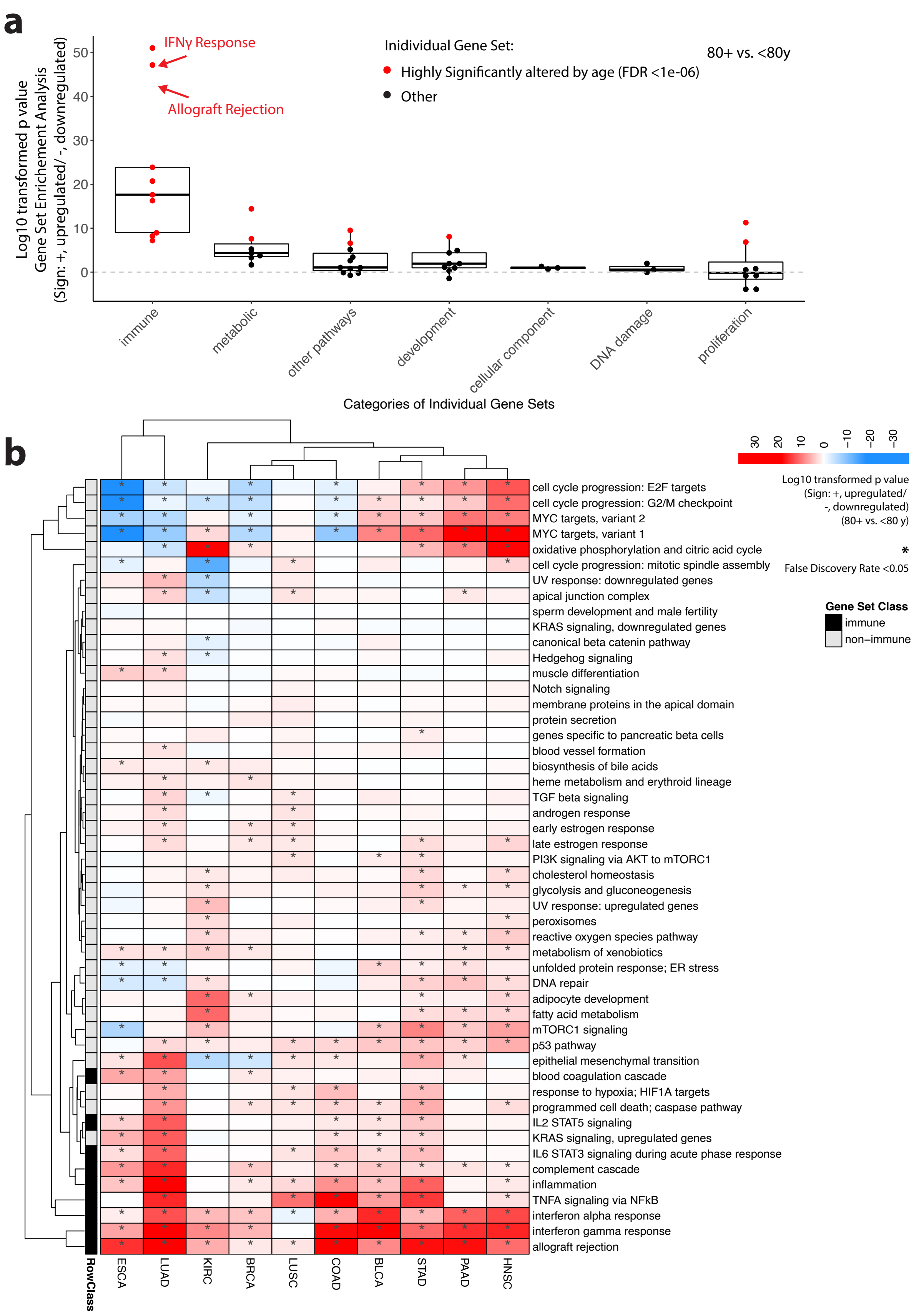
a
IFNγ Response
Allograft Rejection
Inidividual Gene Set:
Highly Significantly altered by age (FDR <1e-06)
Other
80+ vs. <80y
Log10 transformed p value
Gene Set Enrichement Analysis
(Sign: +, upregulated/ -, downregulated)
immune
metabolic
other pathways
development
cellular component
DNA damage
proliferation
Categories of Individual Gene Sets
b
Log10 transformed p value
(Sign: +, upregulated/
-, downregulated)
(80+ vs. <80 y)
*
False Discovery Rate <0.05
Gene Set Class
immune
non–immune
cell cycle progression: E2F targets
cell cycle progression: G2/M checkpoint
MYC targets, variant 2
MYC targets, variant 1
oxidative phosphorylation and citric acid cycle
cell cycle progression: mitotic spindle assembly
UV response: downregulated genes
apical junction complex
sperm development and male fertility
KRAS signaling, downregulated genes
canonical beta catenin pathway
Hedgehog signaling
muscle differentiation
Notch signaling
membrane proteins in the apical domain
protein secretion
genes specific to pancreatic beta cells
blood vessel formation
biosynthesis of bile acids
heme metabolism and erythroid lineage
TGF beta signaling
androgen response
early estrogen response
late estrogen response
PI3K signaling via AKT to mTORC1
cholesterol homeostasis
glycolysis and gluconeogenesis
UV response: upregulated genes
peroxisomes
reactive oxygen species pathway
metabolism of xenobiotics
unfolded protein response; ER stress
DNA repair
adipocyte development
fatty acid metabolism
mTORC1 signaling
p53 pathway
epithelial mesenchymal transition
blood coagulation cascade
response to hypoxia; HIF1A targets
programmed cell death; caspase pathway
IL2 STAT5 signaling
KRAS signaling, upregulated genes
IL6 STAT3 signaling during acute phase response
complement cascade
inflammation
TNFA signaling via NFkB
interferon alpha response
interferon gamma response
allograft rejection
RowClass
ESCA
LUAD
KIRC
BRCA
LUSC
COAD
BLCA
STAD
PAAD
HNSC

Figure S5

a

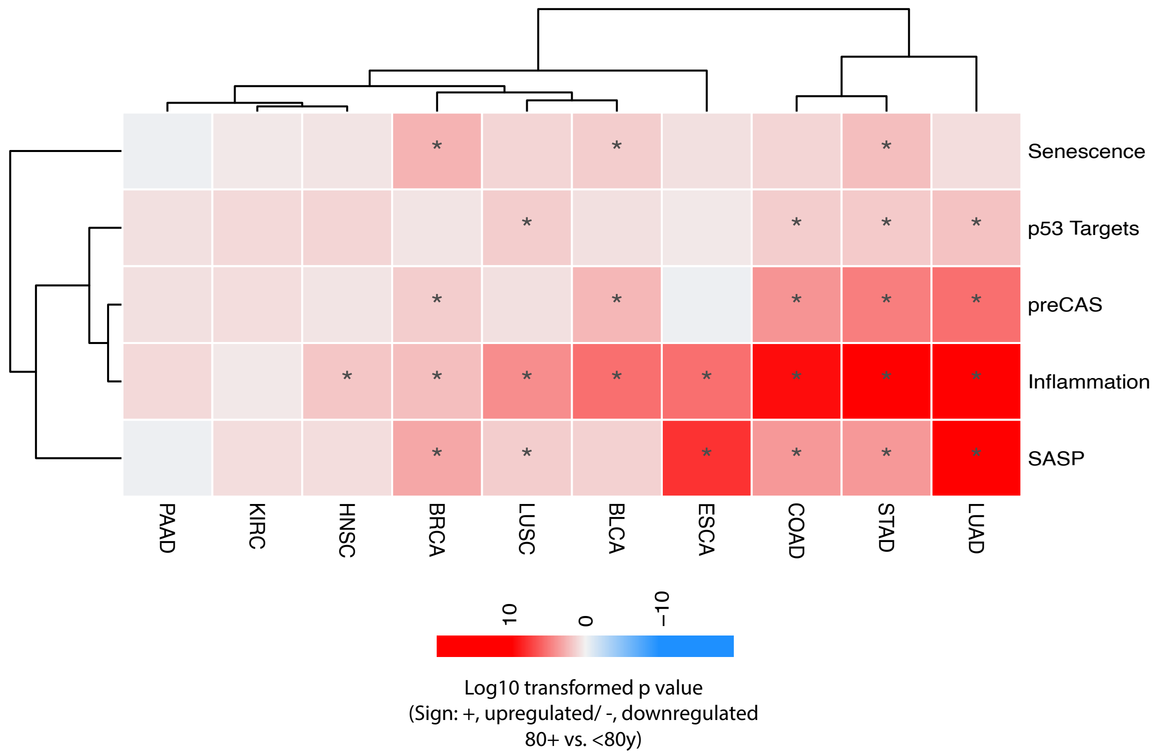
Senescence
p53 Targets
preCAS
Inflammation
SASP
PAAD
KIRC
HNSC
BRCA
LUSC
BLCA
ESCA
COAD
STAD
LUAD
10
0
−10
Log10 transformed p value
(Sign: +, upregulated/ -, downregulated
80+ vs. <80y)

b

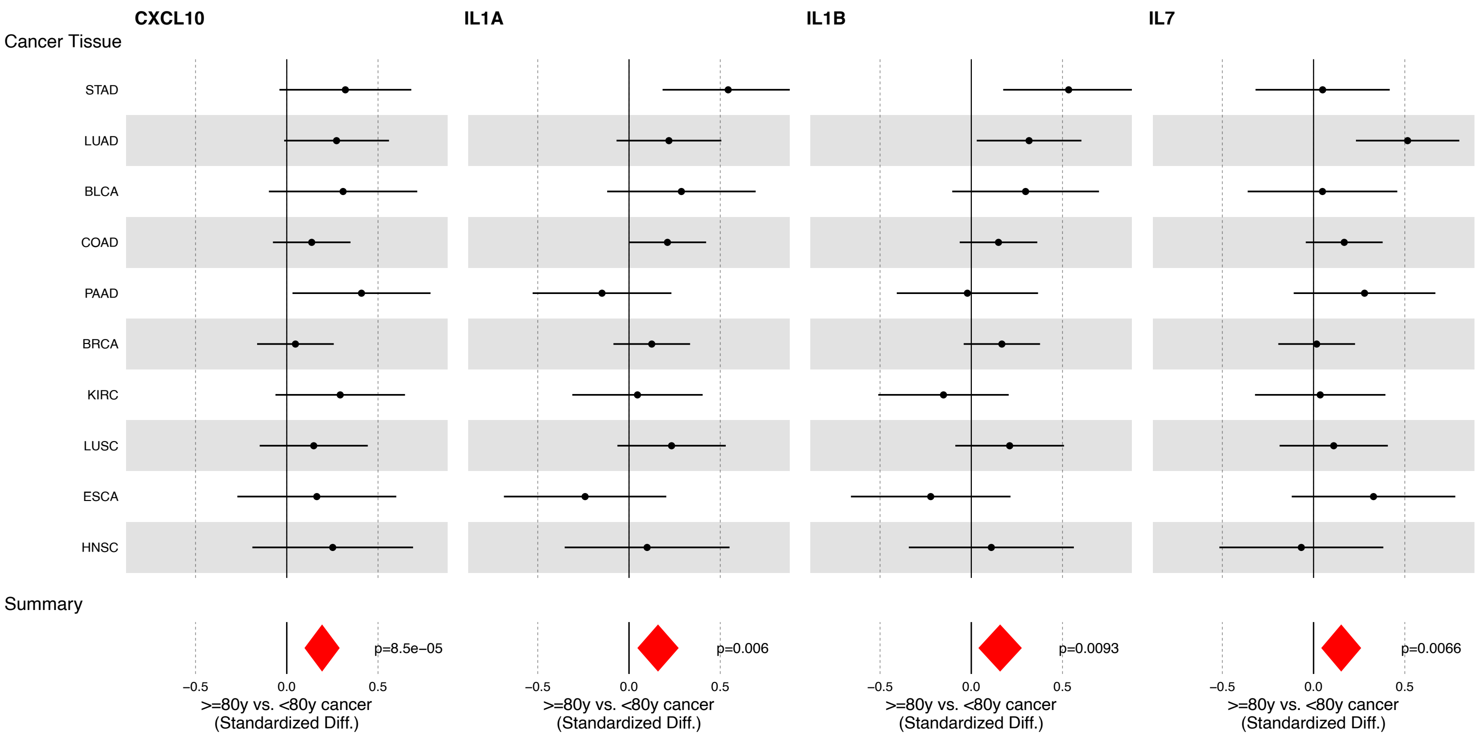
CXCL10
IL1A
IL1B
IL7
Cancer Tissue
STAD
LUAD
BLCA
COAD
PAAD
BRCA
KIRC
LUSC
ESCA
HNSC
Summary
p=8.5e−05
p=0.006
p=0.0093
p=0.0066
−0.5
0.0
0.5
>=80y vs. <80y cancer
(Standardized Diff.)

Figure S6

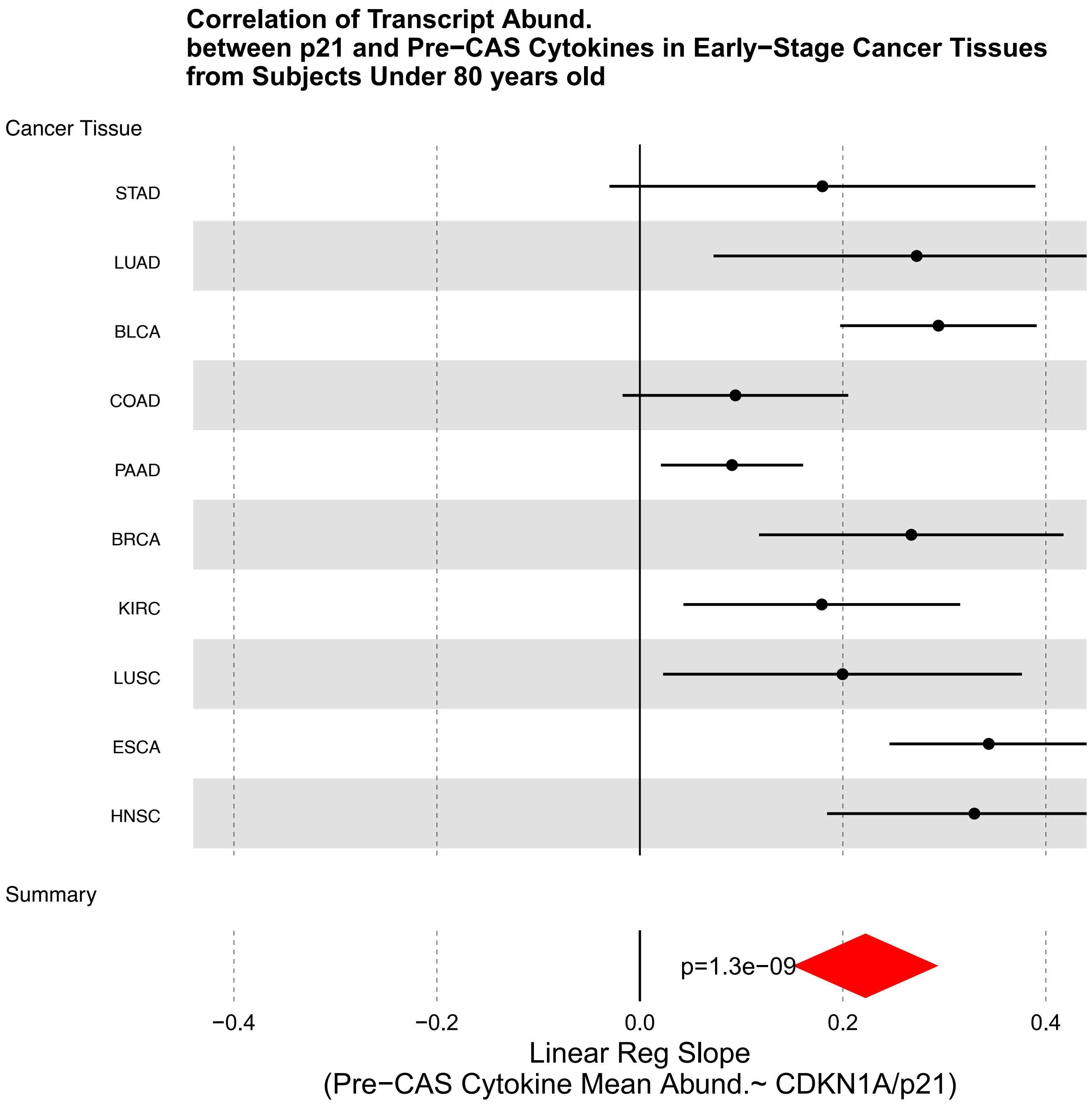
Correlation of Transcript Abund.
between p21 and Pre−CAS Cytokines in Early−Stage Cancer Tissues
from Subjects Under 80 years old
Cancer Tissue
STAD
LUAD
BLCA
COAD
PAAD
BRCA
KIRC
LUSC
ESCA
HNSC
Summary
p=1.3e−09
−0.4
−0.2
0.0
0.2
0.4
Linear Reg Slope
(Pre−CAS Cytokine Mean Abund.~ CDKN1A/p21)

Figure S7

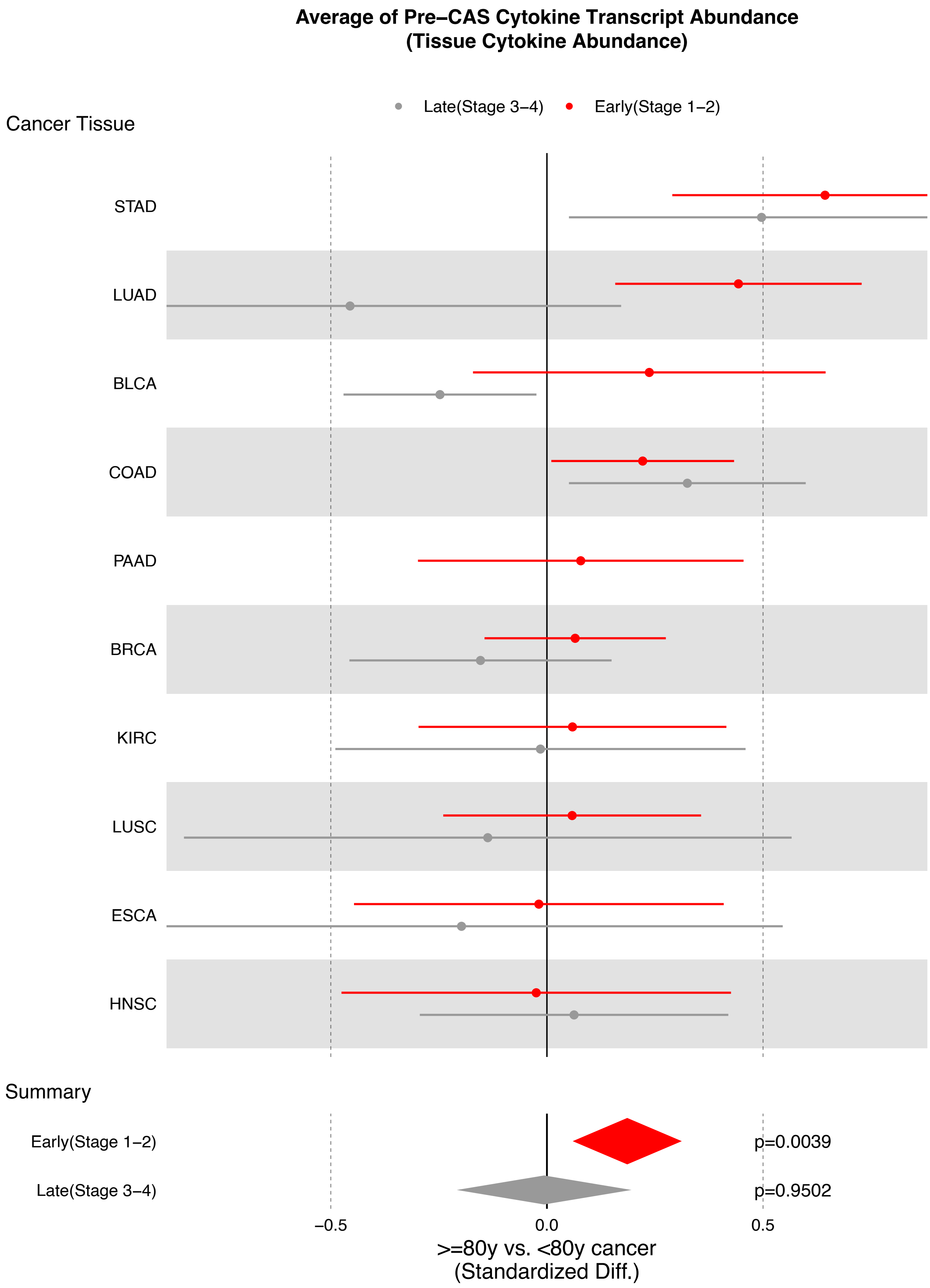
Average of Pre–CAS Cytokine Transcript Abundance
(Tissue Cytokine Abundance)
Late(Stage 3–4)
Early(Stage 1–2)
Cancer Tissue
STAD
LUAD
BLCA
COAD
PAAD
BRCA
KIRC
LUSC
ESCA
HNSC
Summary
Early(Stage 1–2)
p=0.0039
Late(Stage 3–4)
p=0.9502
−0.5
0.0
0.5
>=80y vs. <80y cancer
(Standardized Diff.)

Table S1: Demogrphics

		Enrollment age >=60 y	
	All Subjects	cancer incid. during study*	cancer naive
subject N	133	28	44
sample N	557	154	223
age (at enrollment)	61 (26-80)	80 (74-84)	79 (65-85)
gender (Female%)	58% (77/133)	61% (17/28)	64% (28/44)
follow-up (years)	5 (2-8)	8 (5-8)	7 (4.8-8)

*: In the cohort, the youngest person with a cancer incidence during the study enrolled at the age of 61.

The numbers in the bracket denotes the quantile range (25% - 75%)

Table S2: Cancer incidence

disease	subject N	incidence N	age at incidence(y)*
BCC	14	20	83 (77-87)
SCC	10	11	82 (78-89)
Melanoma	3	5	72 (71-75)
Non-melanoma skin cancer (other)	2	2	90 (90-91)
Bladder Cancer	1	1	74
Lung Cancer	1	1	77
Ovarian Cancer	1	1	89
Colon Cancer	1	1	68
Pancreatic Cancer	1	1	89
Hepatic epithelioid hemangioendothelioma	1	1	77
Ampullary Adenocarcinoma,Gall blader cancer #	1	1	76
Prostate Cancer	1	1	85

*: formatted as median(25%-75%)

#: Two clinical events occurred in the same year on the same subject

Tabel S3: Inflammatory condition incidence

disease	subject N	incidence N	age at incidence(y)*
Dermatitis	5	6	73 (68-78)
Plantar Fasciitis	3	4	74 (68-80)
COPD	3	3	88 (78-88)
Arthritis	3	3	90 (87-96)
Gout	1	1	87
Pancreatitis	1	1	69
Gastritis	1	1	72
Systemic inflammatory response syndrome (SIRS)	1	1	84
Rosacea	1	1	85
Bronchiectasis	1	1	88
Lichen Planus	1	1	68
Osteoarthitis	1	1	74
Pericarditis	1	1	77

*: formatted as median(25%-75%)

Tabel S4: Cardiovascular diseases incidence

disease	subject N	incidence N	age at incidence(y)*
Stroke	5	5	87 (87-91)
Congestive Heart Failure (CHF)	4	4	87 (83-90)
Peripheral Vascular Disease (PVD)	2	3	84 (84-84)
CHF exacerbation	2	2	86 (81-90)
CHF exacerbation,Congestive Heart Failure (CHF)#	1	1	>=90
Myocardial Infraction	1	1	>=90
Angina Pectoris	1	1	75
Transient Ischemic Attack (TIA)	1	1	77
Thalamic Stroke	1	1	79

*: formatted as median(25%-75%)

#: Two clinical events occurred in the same year on the same subject

Table S5: Category for Hallmark Gene Sets

Gene Set ID	Description	N of Genes	Category*	Immune Related
HALLMARK_APICAL_JUNCTION	apical junction complex	200	cellular component	non-immune
HALLMARK_PEROXISOME	peroxisomes	107	cellular component	non-immune
HALLMARK_APICAL_SURFACE	membrane proteins in the apical domain	44	cellular component	non-immune
HALLMARK_ADIPOGENESIS	adipocyte development	200	development	non-immune
HALLMARK_EPITHELIAL_MESENCHYMAL_TRANSITION	epithelial mesenchymal transition	200	development	non-immune
HALLMARK_MYOGENESIS	muscle differentiation	200	development	non-immune
HALLMARK_ESTROGEN_RESPONSE_EARLY	early estrogen response	200	development	non-immune
HALLMARK_ESTROGEN_RESPONSE_LATE	late estrogen response	200	development	non-immune
HALLMARK_SPERMATOGENESIS	sperm development and male fertility	135	development	non-immune
HALLMARK_ANDROGEN_RESPONSE	androgen response	117	development	non-immune
HALLMARK_PANCREAS_BETA_CELLS	genes specific to pancreatic beta cells	40	development	non-immune
HALLMARK_ANGIOGENESIS	blood vessel formation	36	development	non-immune
HALLMARK_UV_RESPONSE_UP	UV response: upregulated genes	158	DNA damage	non-immune
HALLMARK_DNA_REPAIR	DNA repair	150	DNA damage	non-immune
HALLMARK_UV_RESPONSE_DN	UV response: downregulated genes	144	DNA damage	non-immune
HALLMARK_ALLOGRAFT_REJECTION	allograft rejection	200	immune	immune
HALLMARK_COMPLEMENT	complement cascade	200	immune	immune
HALLMARK_INTERFERON_GAMMA_RESPONSE	interferon gamma response	200	immune	immune
HALLMARK_INFLAMMATORY_RESPONSE	inflammation	200	immune	immune
HALLMARK_IL2_STAT5_SIGNALING	IL2 STAT5 signaling	200	immune	immune
HALLMARK_TNFA_SIGNALING_VIA_NFKB	TNFA signaling via NFkB	200	immune	immune
HALLMARK_COAGULATION	blood coagulation cascade	138	immune	immune
HALLMARK_INTERFERON_ALPHA_RESPONSE	interferon alpha response	97	immune	immune
HALLMARK_IL6_JAK_STAT3_SIGNALING	IL6 STAT3 signaling during acute phase response	87	immune	immune
HALLMARK_GLYCOLYSIS	glycolysis and gluconeogenesis	200	metabolic	non-immune
HALLMARK_HEME_METABOLISM	heme metabolism and erythroid lineage	200	metabolic	non-immune
HALLMARK_OXIDATIVE_PHOSPHORYLATION	oxidative phosphorylation and citric acid cycle	200	metabolic	non-immune
HALLMARK_XENOBIOTIC_METABOLISM	metabolism of xenobiotics	200	metabolic	non-immune
HALLMARK_FATTY_ACID_METABOLISM	fatty acid metabolism	158	metabolic	non-immune
HALLMARK_BILE_ACID_METABOLISM	biosynthesis of bile acids	112	metabolic	non-immune
HALLMARK_CHOLESTEROL_HOMEOSTASIS	cholesterol homeostasis	74	metabolic	non-immune
HALLMARK_HYPOXIA	response to hypoxia; HIF1A targets	200	other pathways	non-immune
HALLMARK_MTORC1_SIGNALING	mTORC1 signaling	200	other pathways	non-immune
HALLMARK_APOPTOSIS	programmed cell death; caspase pathway	161	other pathways	non-immune
HALLMARK_UNFOLDED_PROTEIN_RESPONSE	unfolded protein response; ER stress	113	other pathways	non-immune
HALLMARK_PI3K_AKT_MTOR_SIGNALING	PI3K signaling via AKT to mTORC1	105	other pathways	non-immune
HALLMARK_PROTEIN_SECRETION	protein secretion	96	other pathways	non-immune
HALLMARK_TGF_BETA_SIGNALING	TGF beta signaling	54	other pathways	non-immune
HALLMARK_REACTIVE_OXYGEN_SPECIES_PATHWAY	reactive oxygen species pathway	49	other pathways	non-immune
HALLMARK_WNT_BETA_CATENIN_SIGNALING	canonical beta catenin pathway	42	other pathways	non-immune
HALLMARK_HEDGEHOG_SIGNALING	Hedgehog signaling	36	other pathways	non-immune
HALLMARK_NOTCH_SIGNALING	Notch signaling	32	other pathways	non-immune
HALLMARK_E2F_TARGETS	cell cycle progression: E2F targets	200	proliferation	non-immune
HALLMARK_G2M_CHECKPOINT	cell cycle progression: G2/M checkpoint	200	proliferation	non-immune
HALLMARK_MYC_TARGETS_V1	MYC targets, variant 1	200	proliferation	non-immune
HALLMARK_P53_PATHWAY	p53 pathway	200	proliferation	non-immune
HALLMARK_MITOTIC_SPINDLE	cell cycle progression: mitotic spindle assembly	200	proliferation	non-immune
HALLMARK_KRAS_SIGNALING_UP	KRAS signaling, upregulated genes	200	proliferation	non-immune
HALLMARK_KRAS_SIGNALING_DN	KRAS signaling, downregulated genes	200	proliferation	non-immune
HALLMARK_MYC_TARGETS_V2	MYC targets, variant 2	58	proliferation	non-immune

*: modified from Liberzon A et. al.: The Molecular Signatures Database Hallmark Gene Set Collection. Cell systems 2015, 1(6):417-425.
In the original categorization, several immune (IL2 STAT5 and TNF signaling), proliferation and development related gene sets are categorized under a non-specific term "pathway" or "signaling". These gene sets are re-assigned to an according category now.

Table S6: Hallmark Gene Sets Changed by an Advanced Age in Early-stage Tissues Across Cancer Types (>80y vs. 80+y)

Gene Set ID	Description	Category	Activity Change	False Discovery Rate
HALLMARK_ALLOGRAFT_REJECTION	allograft rejection	immune	Highly Significantly UP-regulated	9.30E-52
HALLMARK_INTERFERON_GAMMA_RESPONSE	interferon gamma response	immune	Highly Significantly UP-regulated	6.90E-48
HALLMARK_TNFA_SIGNALING_VIA_NFKB	TNFA signaling via NFkB	immune	Highly Significantly UP-regulated	1.40E-24
HALLMARK_INTERFERON_ALPHA_RESPONSE	interferon alpha response	immune	Highly Significantly UP-regulated	1.80E-21
HALLMARK_INFLAMMATORY_RESPONSE	inflammation	immune	Highly Significantly UP-regulated	2.30E-18
HALLMARK_COMPLEMENT	complement cascade	immune	Highly Significantly UP-regulated	5.10E-17
HALLMARK_OXIDATIVE_PHOSPHORYLATION	oxidative phosphorylation and citric acid cycle	metabolic	Highly Significantly UP-regulated	3.80E-15
HALLMARK_P53_PATHWAY	p53 pathway	proliferation	Highly Significantly UP-regulated	5.20E-12
HALLMARK_APOPTOSIS	programmed cell death; caspase pathway	other pathways	Highly Significantly UP-regulated	3.00E-10
HALLMARK_IL6_JAK_STAT3_SIGNALING	IL6 STAT3 signaling during acute phase response	immune	Highly Significantly UP-regulated	1.00E-09
HALLMARK_IL2_STAT5_SIGNALING	IL2 STAT5 signaling	immune	Highly Significantly UP-regulated	5.90E-09
HALLMARK_ADIPOGENESIS	adipocyte development	development	Highly Significantly UP-regulated	8.70E-09
HALLMARK_FATTY_ACID_METABOLISM	fatty acid metabolism	metabolic	Highly Significantly UP-regulated	2.60E-08
HALLMARK_COAGULATION	blood coagulation cascade	immune	Highly Significantly UP-regulated	6.30E-08
HALLMARK_KRAS_SIGNALING_UP	KRAS signaling, upregulated genes	proliferation	Highly Significantly UP-regulated	1.40E-07
HALLMARK_HYPOXIA	response to hypoxia; HIF1A targets	other pathways	Highly Significantly UP-regulated	2.50E-07
HALLMARK_XENOBIOTIC_METABOLISM	metabolism of xenobiotics	metabolic	Significantly UP-regulated	5.70E-06
HALLMARK_MTORC1_SIGNALING	mTORC1 signaling	other pathways	Significantly UP-regulated	6.80E-06
HALLMARK_ESTROGEN_RESPONSE_LATE	late estrogen response	development	Significantly UP-regulated	1.20E-05
HALLMARK_ESTROGEN_RESPONSE_EARLY	early estrogen response	development	Significantly UP-regulated	3.90E-05
HALLMARK_GLYCOLYSIS	glycolysis and gluconeogenesis	metabolic	Significantly UP-regulated	4.40E-05
HALLMARK_E2F_TARGETS	cell cycle progression: E2F targets	proliferation	Significantly DN-regulated	0.00013
HALLMARK_G2M_CHECKPOINT	cell cycle progression: G2/M checkpoint	proliferation	Significantly DN-regulated	0.00013
HALLMARK_CHOLESTEROL_HOMEOSTASIS	cholesterol homeostasis	metabolic	Significantly UP-regulated	0.00017
HALLMARK_REACTIVE_OXYGEN_SPECIES_PATHWAY	reactive oxygen species pathway	other pathways	Significantly UP-regulated	0.00037
HALLMARK_HEME_METABOLISM	heme metabolism and erythroid lineage	metabolic	Significantly UP-regulated	0.00049
HALLMARK_PI3K_AKT_MTOR_SIGNALING	PI3K signaling via AKT to mTORC1	other pathways	Significantly UP-regulated	0.0023
HALLMARK_UV_RESPONSE_UP	UV response: upregulated genes	DNA damage	Significantly UP-regulated	0.01
HALLMARK_EPITHELIAL_MESENCHYMAL_TRANSITION	epithelial mesenchymal transition	development	Significantly UP-regulated	0.011
HALLMARK_ANDROGEN_RESPONSE	androgen response	development	Significantly UP-regulated	0.012
HALLMARK_BILE_ACID_METABOLISM	biosynthesis of bile acids	metabolic	Significantly UP-regulated	0.021
HALLMARK_SPERMATOGENESIS	sperm development and male fertility	development	Significantly DN-regulated	0.037
HALLMARK_PEROXISOME	peroxisomes	cellular component	Significantly UP-regulated	0.047
HALLMARK_ANGIOGENESIS	blood vessel formation	development	Not Significantly Changed	0.071
HALLMARK_PROTEIN_SECRETION	protein secretion	other pathways	Not Significantly Changed	0.086
HALLMARK_TGF_BETA_SIGNALING	TGF beta signaling	other pathways	Not Significantly Changed	0.094
HALLMARK_APICAL_SURFACE	membrane proteins in the apical domain	cellular component	Not Significantly Changed	0.1
HALLMARK_PANCREAS_BETA_CELLS	genes specific to pancreatic beta cells	development	Not Significantly Changed	0.1
HALLMARK_MITOTIC_SPINDLE	cell cycle progression: mitotic spindle assembly	proliferation	Not Significantly Changed	0.15
HALLMARK_NOTCH_SIGNALING	Notch signaling	other pathways	Not Significantly Changed	0.16
HALLMARK_KRAS_SIGNALING_DN	KRAS signaling, downregulated genes	proliferation	Not Significantly Changed	0.16
HALLMARK_MYC_TARGETS_V1	MYC targets, variant 1	proliferation	Not Significantly Changed	0.17
HALLMARK_WNT_BETA_CATENIN_SIGNALING	canonical beta catenin pathway	other pathways	Not Significantly Changed	0.2
HALLMARK_APICAL_JUNCTION	apical junction complex	cellular component	Not Significantly Changed	0.21
HALLMARK_DNA_REPAIR	DNA repair	DNA damage	Not Significantly Changed	0.26
HALLMARK_MYC_TARGETS_V2	MYC targets, variant 2	proliferation	Not Significantly Changed	0.3
HALLMARK_MYOGENESIS	muscle differentiation	development	Not Significantly Changed	0.51
HALLMARK_UNFOLDED_PROTEIN_RESPONSE	unfolded protein response; ER stress	other pathways	Not Significantly Changed	0.63
HALLMARK_HEDGEHOG_SIGNALING	Hedgehog signaling	other pathways	Not Significantly Changed	0.82
HALLMARK_UV_RESPONSE_DN	UV response: downregulated genes	DNA damage	Not Significantly Changed	0.91

References

1. A. Alpert *et al.*, A clinically meaningful metric of immune age derived from high-dimensional longitudinal monitoring. *Nature Medicine* **25**, 487-495 (2019).
2. W. E. Johnson, C. Li, A. Rabinovic, Adjusting batch effects in microarray expression data using empirical Bayes methods. *Biostatistics (Oxford, England)* **8**, 118-127 (2006).
3. J. T. Leek, W. E. Johnson, H. S. Parker, A. E. Jaffe, J. D. Storey, The sva package for removing batch effects and other unwanted variation in high-throughput experiments. *Bioinformatics (Oxford, England)* **28**, 882-883 (2012).
4. D. E. Ho, K. Imai, G. King, E. A. Stuart, Matching as Nonparametric Preprocessing for Reducing Model Dependence in Parametric Causal Inference. *Political Analysis* **15**, 199-236 (2007).
5. D. E. Ho, K. Imai, G. King, E. A. Stuart, MatchIt: Nonparametric Preprocessing for Parametric Causal Inference. *J Stat Softw* **42** (2011).
6. M. E. Ritchie *et al.*, limma powers differential expression analyses for RNA-sequencing and microarray studies. *Nucleic Acids Research* **43**, e47-e47 (2015).
7. K. Chang *et al.*, The Cancer Genome Atlas Pan-Cancer analysis project. *Nature Genetics* **45**, 1113-1120 (2013).
8. C. J. Lortie, A. Filazzola, A contrast of meta and metafor packages for meta-analyses in R. *Ecology and Evolution* **10**, 10916-10921 (2020).
9. A. Liberzon *et al.*, The Molecular Signatures Database Hallmark Gene Set Collection. *Cell systems* **1**, 417-425 (2015).
10. W. Luo, M. S. Friedman, K. Shedden, K. D. Hankenson, P. J. Woolf, GAGE: generally applicable gene set enrichment for pathway analysis. *BMC Bioinformatics* **10**, 161 (2009).
